# Supplementary material for: Phosphonic Acid Analogs of Fluorophenylalanines as Inhibitors of Human and Porcine Aminopeptidases N: Validation of the Importance of the Substitution of the Aromatic Ring
Source: Biomolecules. 2020 Apr 9;10(4):579. doi: 10.3390/biom10040579 (PMC7226027; doi:10.3390/biom10040579)
Supplement: Supplementary file 1 [file biomolecules-10-00579-s001.zip › Supplementary_Material.pdf]

biomolecules

Article

Phosphonic Acid Analogs of Fluorophenylalanines as Inhibitors of Human and Porcine Aminopeptidases N: Validation of the Importance of the Substitution of Aromatic Ring

Supplementary Materials:

Below, we have shown structures and NMR spectra (^{1}H and ^{31}P NMR) for representative tautomeric mixture of ketophosphonate (**3l**) and enolphosphonate (**4l**) obtained upon synthesis of *diethyl 2-(3,4,5-trifluorophenyl)ethylphosphonate* (**Figure S1**). **Figure S2** present geometric isomerization during oxime formation detectable by NMR spectra (^{1}H (**A**), ^{19}F (**B**), ^{31}P (**C**) NMR) for *diethyl (E/Z)-1-hydroxyimino-2-(3,4,5-trifluorophenyl)ethylphosphonate* (**5l**) (representative example). **Figure S3** showed complete conversion of *E*/*Z* isomeric mixture (**5l**, 54:46) after a time into presumable *Z*-isomer, recorded by NMR (^{1}H (**A**), ^{19}F (**B**), ^{31}P (**C**) NMR) of this same compound from *E*/*Z* isomers mixture to only *Z form* included. **Figure S4** is focused on showing dehalogenation in phenyl ring during reduction of the oxime group to the amine group by using ^{1}H NMR spectra (*diethyl (Z)-1-hydroxyimino-(2-bromo-4-fluorophenyl)ethylphosphonate* was chosen as representative example). NMR spectra for representative final aminophosphonic acids are presented in the **Figures S5** and **S6**. For the representative example *1-amino-2-(3,4-difluorophenyl)ethylphosphonic acid* (**1h**) we have chosen ^{1}H, ^{1}H-^{1}H COSY and 2D ^{1}H-^{31}P HMQC (**Figure S5**) NMR spectra in order to show couplings between proton-proton and proton-phosphorus. **Figure S6A** reveals the overlapping of the aliphatic proton peaks, which results from proximity substituents located in *orto* position in aromatic moiety to aminophosphonate fragment, shown for 1-*amino-2-(2,6-difluorophenyl)ethylphosphonic acid* (**1g**). For this same compound we obtained very low coupling between phosphorus-fluorine, which appears in ^{31}P NMR spectrum (**Figure S6B**). Crystal structure of compounds **7d** is shown in the **Figure S7**. The supplementary **Figures S8** and **S9** included modes of binding of compounds **1t** and **1g** with hAPN and pAPN, respectively.

Figure S1. ^{1}H **(A)** and ^{31}P NMR **(B)** spectra of the keto- (**3l**) and enol- (**4l**) tautomers of diethyl 2-(3,4,5-trifluorophenyl)ethylphosphonate.

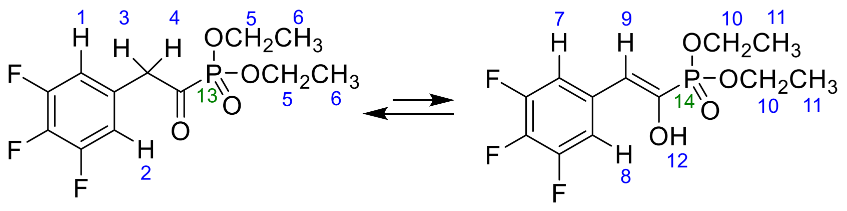

66 : 34

keto *enol*

A

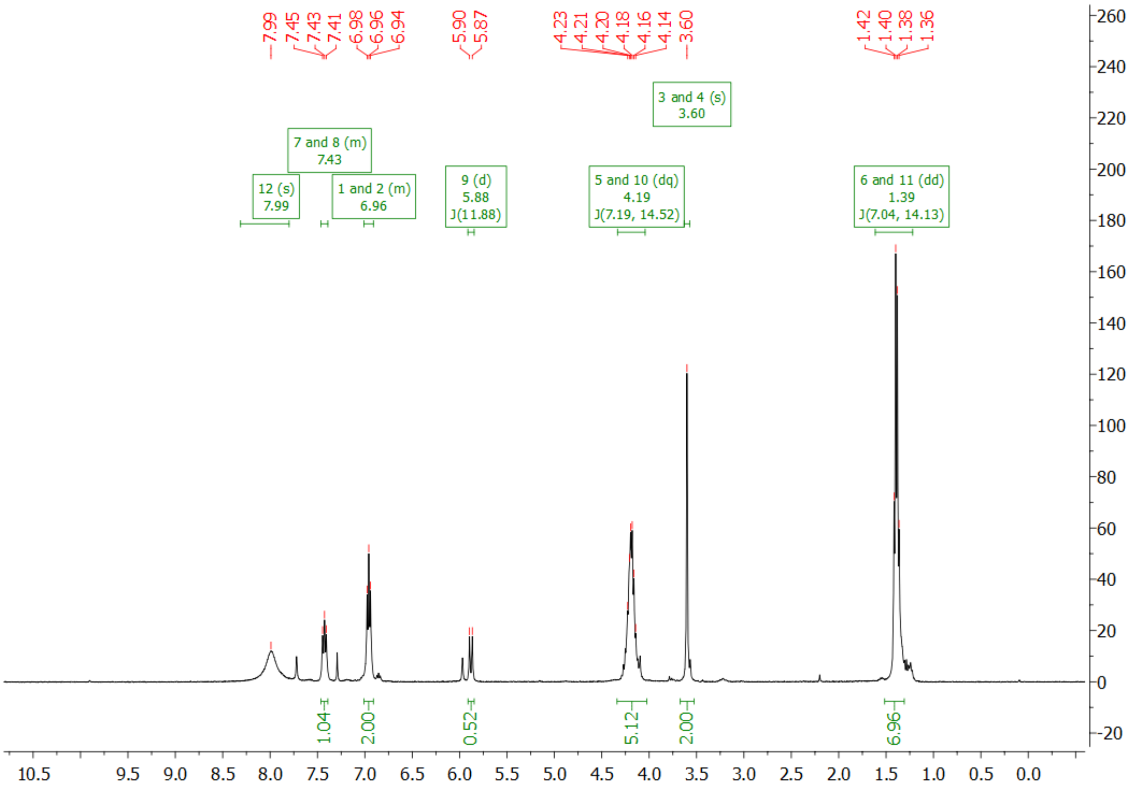

B

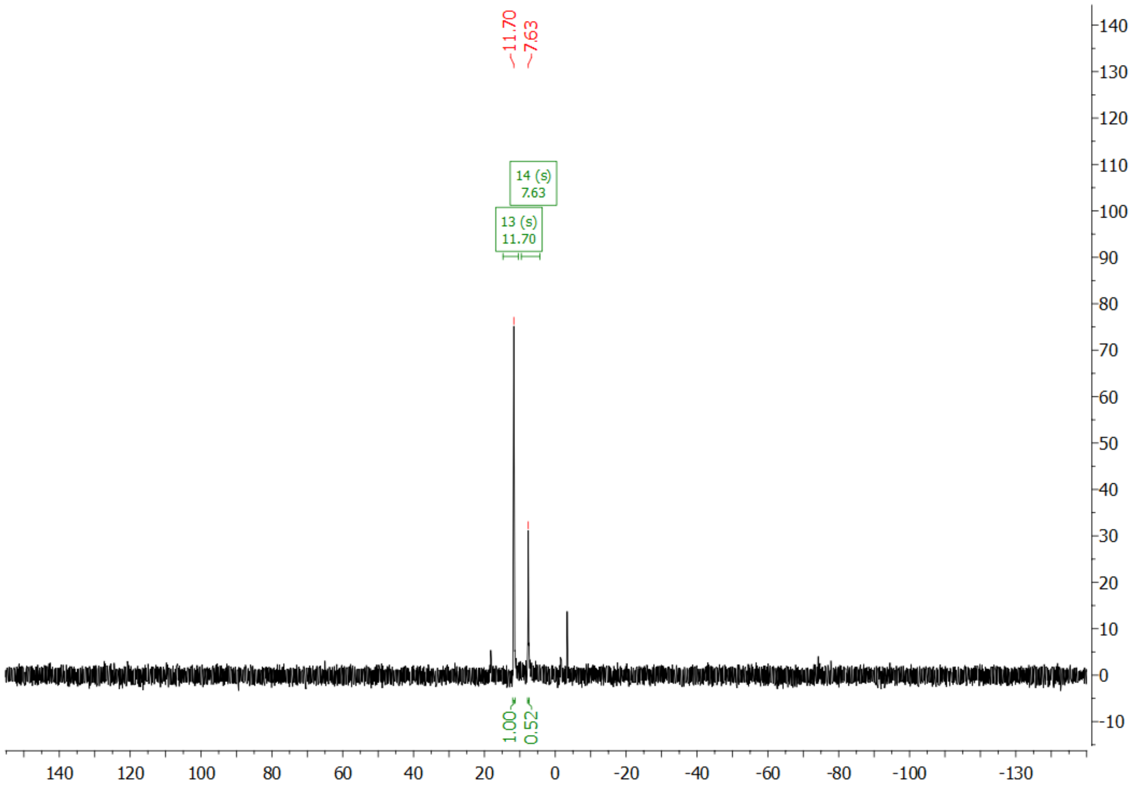

^{1}H NMR (400 MHz, $CDCl_3$) (A), δ= 7.99 (brs, 1H, OH), 7.47 – 7.38 (m, 2H, CH_{ar} enol form), 7.01 – 6.90 (m, 2H, CH_{ar} keto form), 5.88 (d, *J* = 11.9 Hz, 1H, CHP, enol form), 4.19 (dq, *J* = 7.19, 14.52 Hz, 4H, 2xOC**H**$_2$CH$_3$), 3.60 (s, 2H, CH_2, keto form), 1.39 (dd, *J* = 7.04, 14.13 Hz, 6H, 2xOCH$_2$C**H**$_3$) ppm.

^{31}P NMR (162 MHz, $CDCl_3$) (B), δ= 11.70 (s, 1P, keto form), 7.65 (s. 1P, enol form) ppm.

Figure S2. ^{1}H **(A)**, ^{19}F **(B)** and ^{31}P NMR **(C)** spectra of mixture of isomers diethyl (E/Z)-1-hydroxyimino-2-(3,4,5-trifluorophenyl)ethylphosphonate (**5l**).

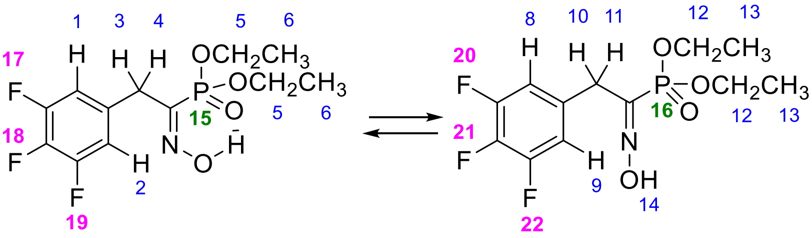

46 : 54

Z form *E form*

A

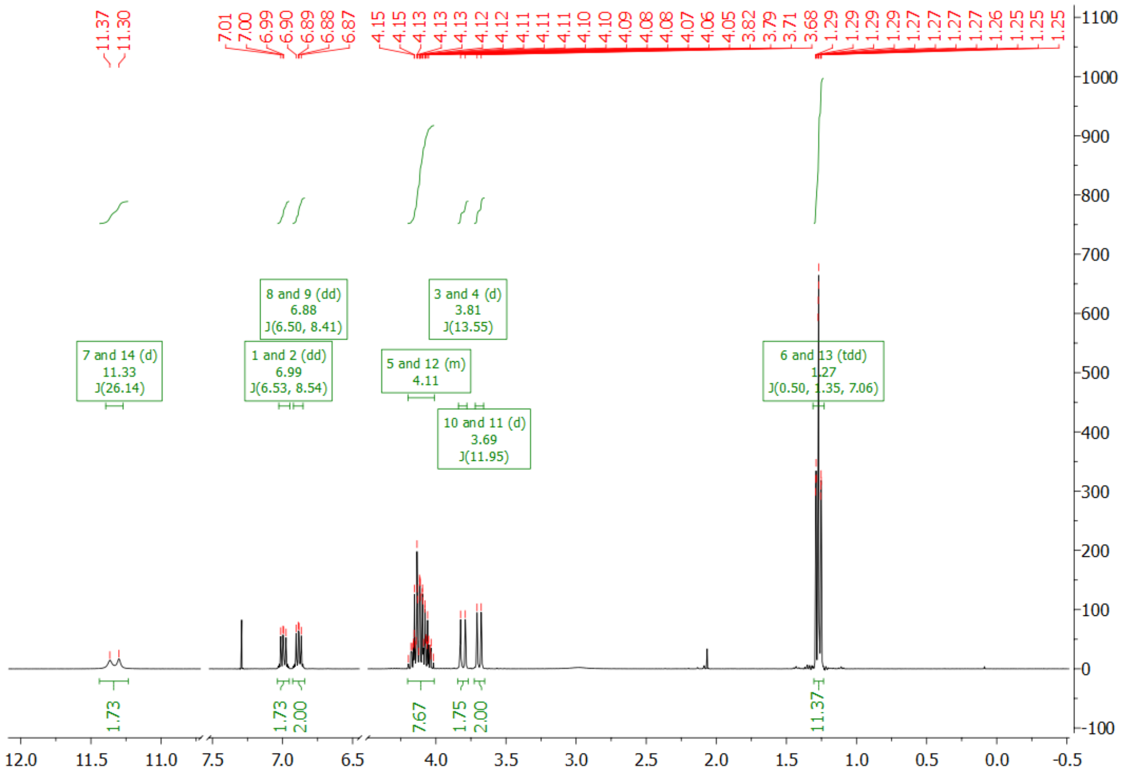

B

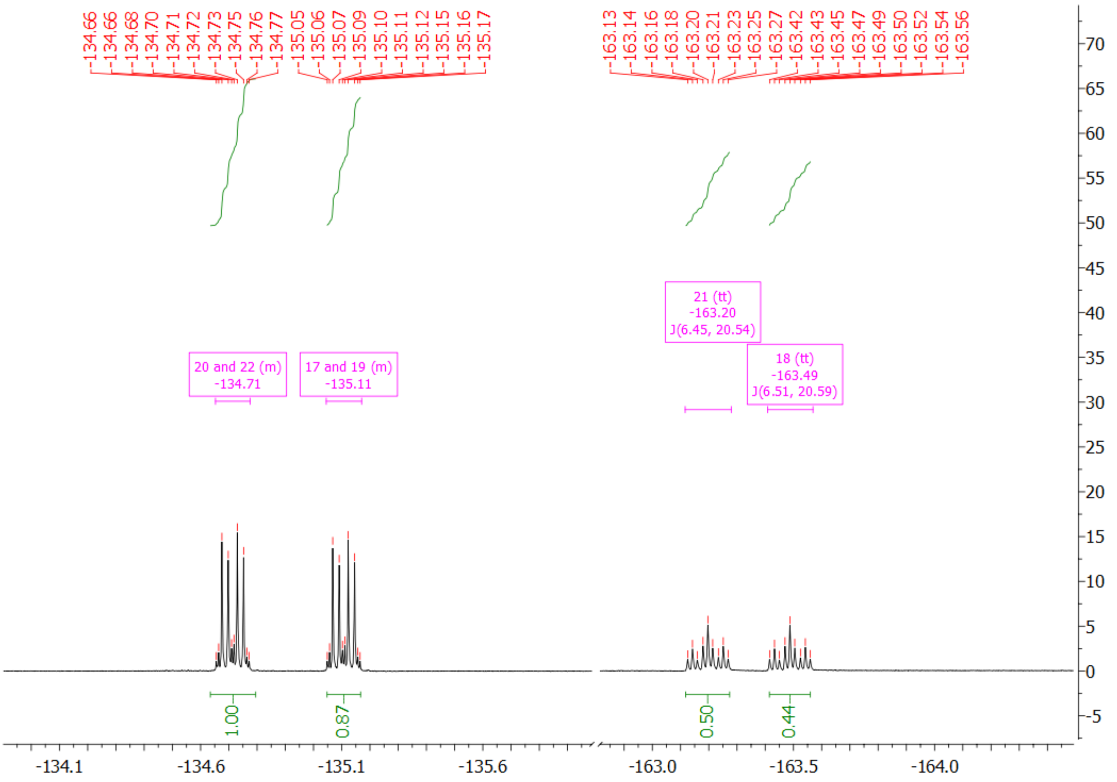

C

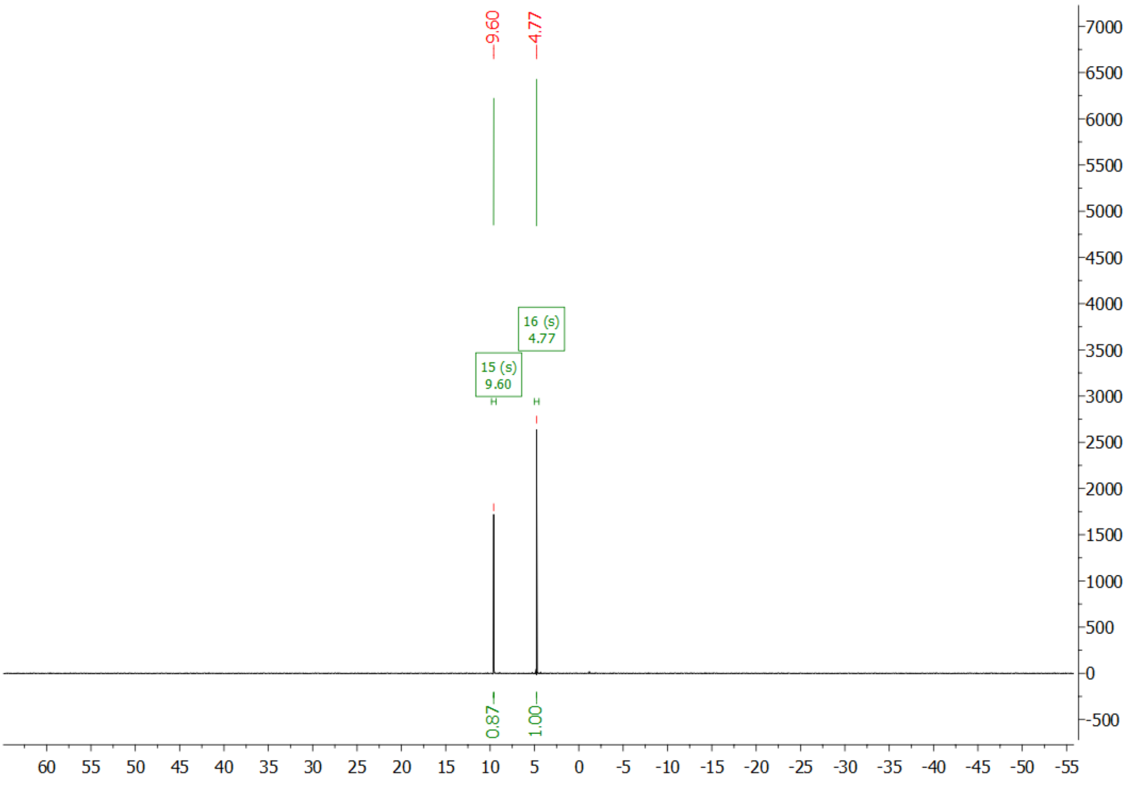

^{1}H NMR (400 MHz, CDCl$_3$), (A): δ = 11.33 (d, *J* = 25.6 Hz, 2H, NOH, E/Z form), 6.99 (dd, *J* = 8.5, 6.5 Hz, 2H, CH$_{ar}$, *Z form*), 6.88 (dd, *J* = 8.4, 6.5 Hz, 2H, CH$_{ar}$, *E form*), 4.22 – 3.99 (m, 4H, 2xOC**H**$_2$CH$_3$), 3.81 (d, *J* = 13.5 Hz, 2H, *Z form*), 3.69 (d, *J* = 11.9 Hz, 2H, *E form*), 1.27 (tdd, *J* = 7.1, 1.4, 0.5 Hz, 6H, 2xOCH$_2$C**H**$_3$) ppm.

^{19}F NMR (377 MHz, CDCl3), (B): δ = -134.65 – -134.78 (m, 2F, *E form*), -135.05 – -135.17 (m, 2F, *Z form*), -163.20 (tt, *J* = 20.5,6.4Hz, 1F, *E form*), -163.49 (tt, *J* = 20.6,6.5Hz, *Z form*) ppm.

^{31}P NMR (162 MHz, CDCl3), (C): δ = 9.60 (s, 1P, *Z form*), 4.77 (s, 1P, *E form*) ppm.

Figure S3. ^{1}H **(A)**, ^{19}F **(B)** and ^{31}P NMR **(C)** spectra of diethyl (Z)-1-hydroxyimino-2-(3,4,5-trifluorophenyl)ethylphosphonate (**5l**) obtained after storage in $CDCl_3$.

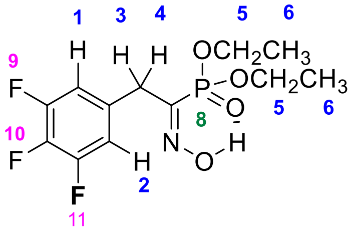

A

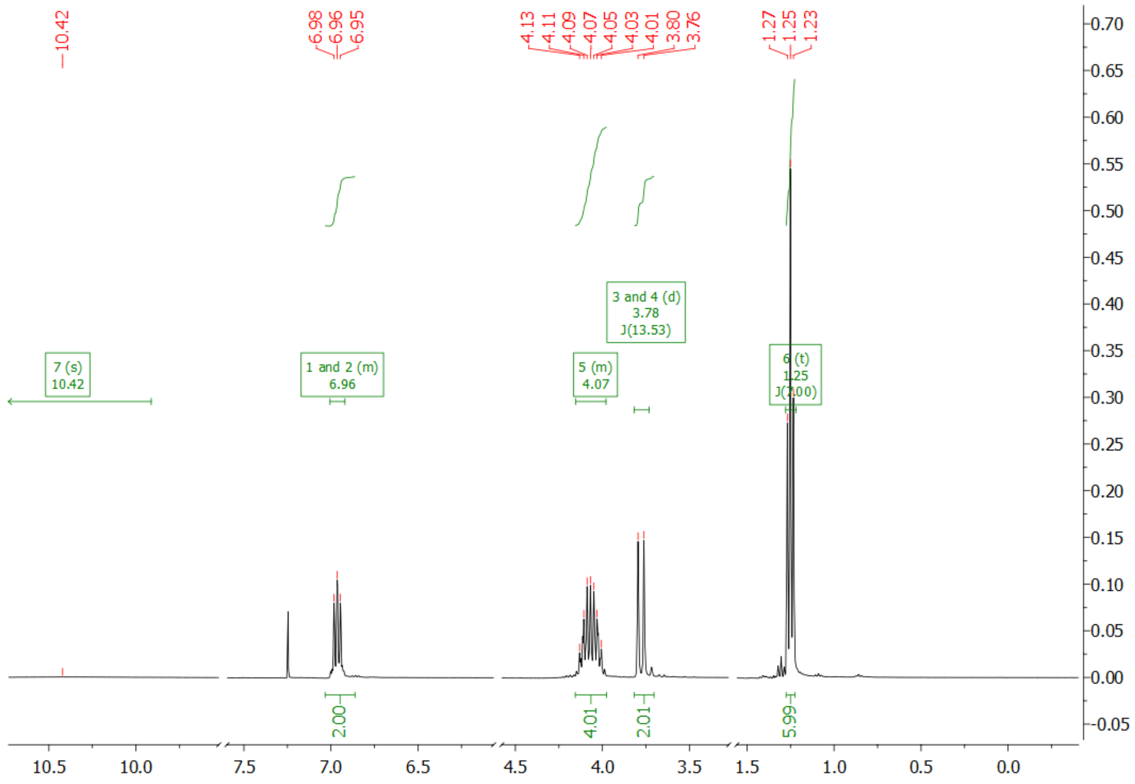

B

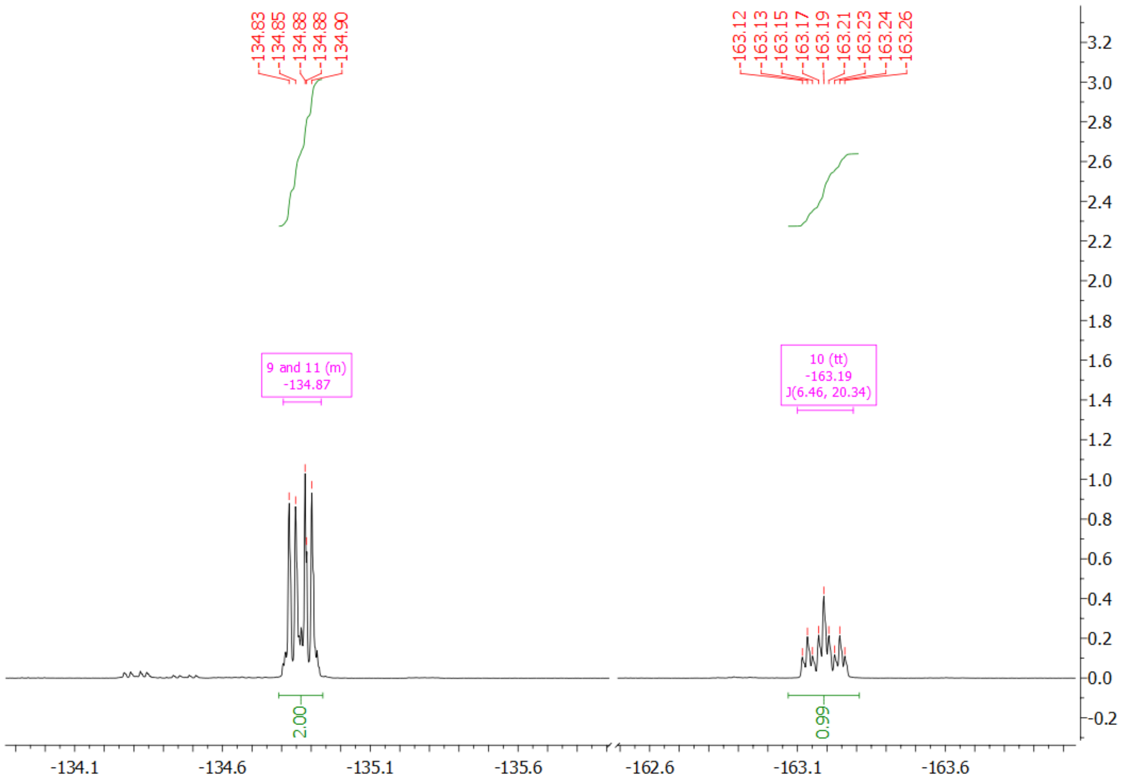

C

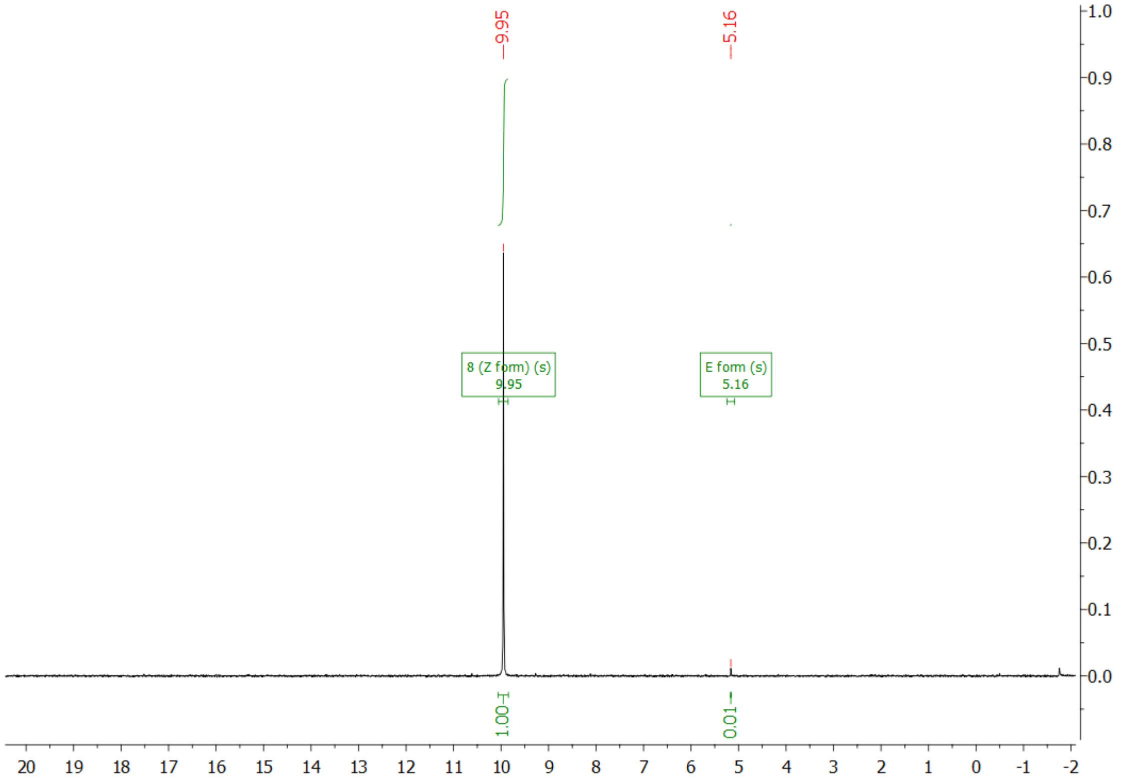

^{1}H NMR (400 MHz, $CDCl_3$), (A): δ = 10.42 (br s, 1H, NOH, *Z form*), 7.01-6.92 (m, 2H, CH_{ar}, *Z form*), 4.15 – 3.98 (m, 4H, 2xOC**H_2**CH_3), 3.78 (d, *J* = 13.5 Hz, 2H, **CH_2**CP *Z form*), 1.25 (t, *J* = 7.1 Hz, 6H, 2xOCH_2**CH_3**) ppm.

^{19}F NMR (377 MHz, CDCl3), (B): δ = -134.81 - -134.93 (m, 2F, *Z form*), -163.19 (tt, *J* = 20.3,6.5 Hz, *Z form*) ppm.

^{31}P NMR (162 MHz, CDCl3), (C): δ = 9.95 (s, 1P, *Z form*) ppm.

Figure S4. ^{1}H NMR spectra of the product of debromination reaction during reduction of the oxime (**1**) to the amine intermediate (**2**). As the respresentative example aromatic region of the diethyl (Z)-1-hydroxyimino-(2-bromo-4-fluorophenyl)ethylphosphonate was shown: (**A**) aromatic fragment of the oxime and (**B**) aromatic fragment of the aminophosphonate product.

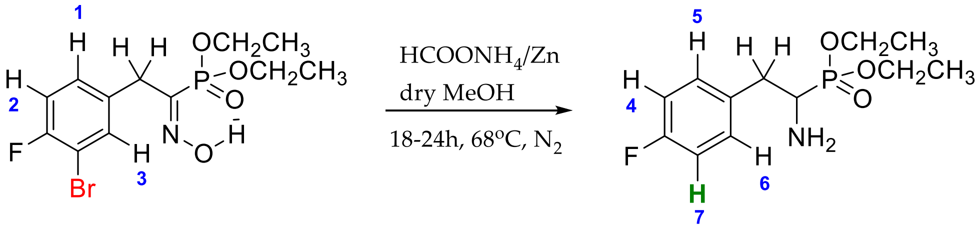

1 **2**

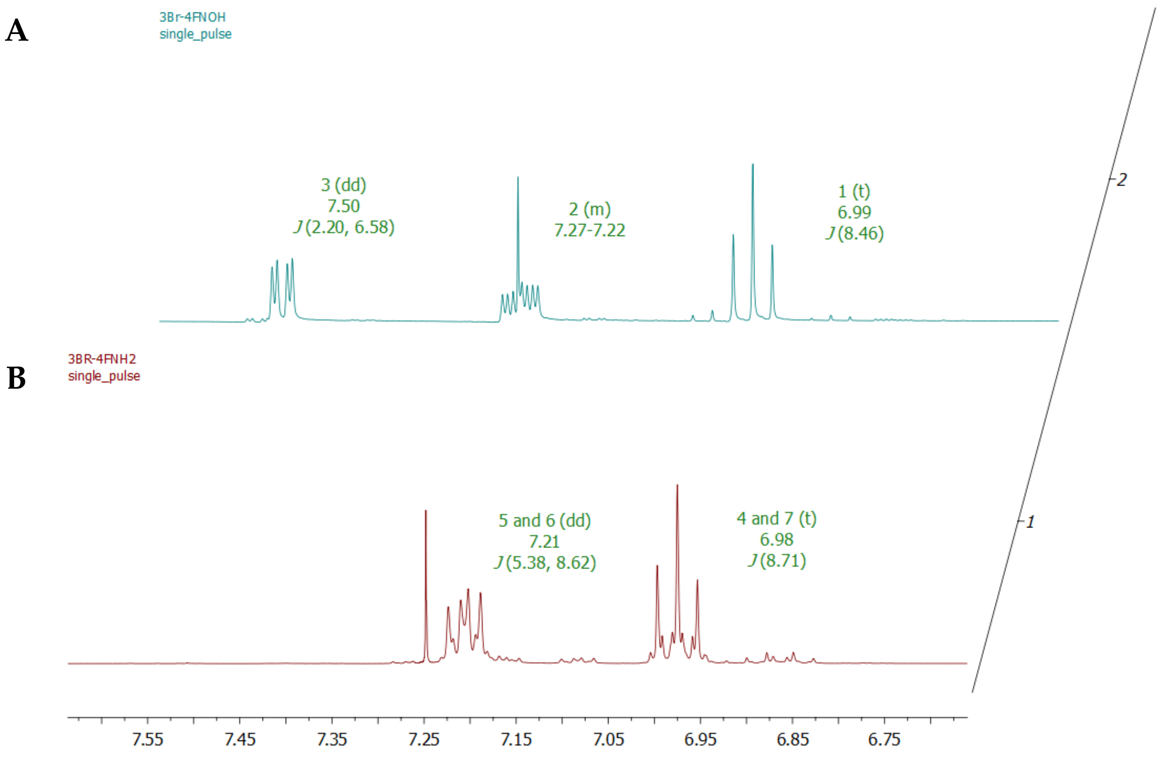

Figure S5. ^{1}H **(A)**, ^{1}H-^{1}H COSY **(B)** and ^{1}H-^{31}P HMQC **(C)** for 1-Amino-2-(3,4-difluorophenyl)ethylphosphonic acid (**1h**).

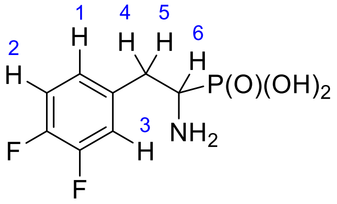

A

B

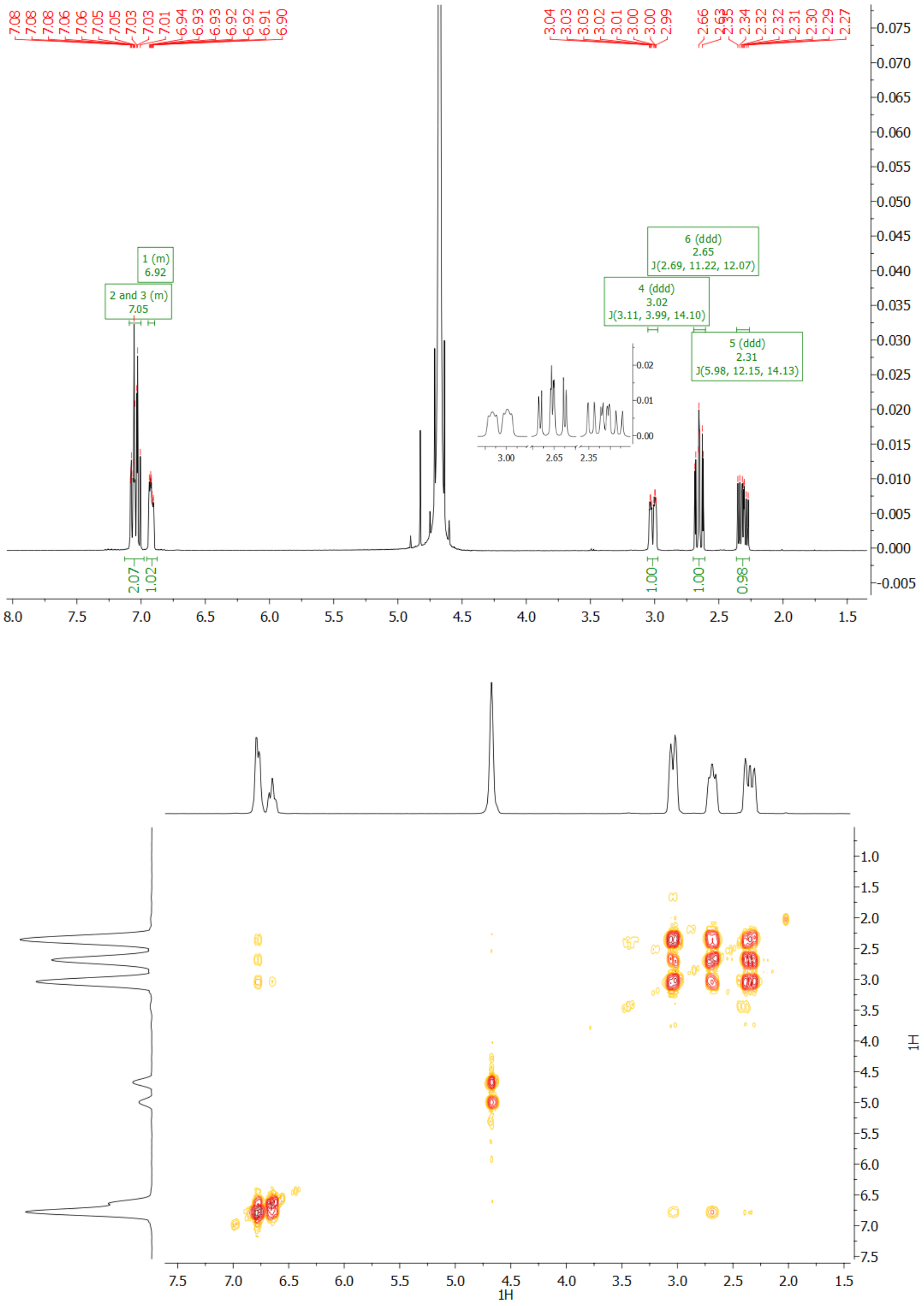
7.08 7.08 7.08 7.06 7.06 7.05 7.05 7.03 7.03 7.01 6.94 6.93 6.93 6.92 6.92 6.91 6.90
3.04 3.03 3.03 3.02 3.01 3.00 3.00 2.99
2.66 2.63 2.35 2.34 2.32 2.32 2.31 2.30 2.29 2.27
1 (m)
6.92
2 and 3 (m)
7.05
6 (ddd)
2.65
J(2.69, 11.22, 12.07)
4 (ddd)
3.02
J(3.11, 3.99, 14.10)
5 (ddd)
2.31
J(5.98, 12.15, 14.13)
2.07
1.02
1.00
1.00
0.98
1H

C

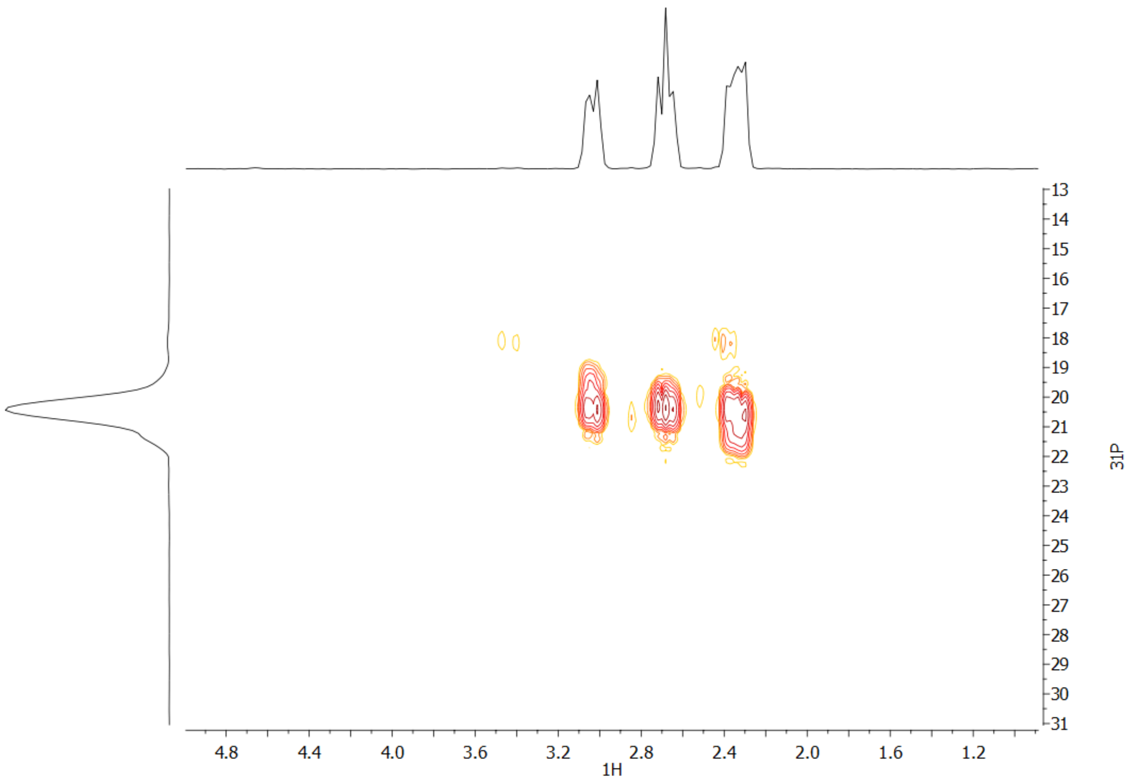

^{1}H NMR (400MHz, D_2O+NaOD), (A): δ, ppm: 7.08 – 7.01 (m, 2H, 2xCH$_{ar}$), 6.82 6.94 – 6.90 (m, 1H, CH$_{ar}$), 4.67 (br s, 1H, NH), 3.02 (ddd, $^2J_{HH}$ = 14.1, $^3J_{HP}$ = 4.0, $^3J_{HH}$ = 3.1 Hz, 1H) and 2.31 (ddd, $^2J_{HH}$ = 14.1, $^3J_{HH}$ = 12.1, $^3J_{HP}$ = 6.0 Hz, 1H) (CH_2), 2.65 (ddd, $^3J_{HH}$ = 12.1, $^2J_{HP}$ = 11.2, $^3J_{HH}$ = 2.7 Hz, 1H, CHP).

^{1}H NMR spectrum of the final aminophosphonic acid shows couplings of the aromatic protons with fluorine substituents and aliphatic protons with phosphorus atom.

^{1}H-^{1}H COSY NMR (B) indicates correlation between aliphatic protons and low couplings between aliphatic-aromatic protons.

^{1}H-^{31}P HMQC (C) shows correlations between aliphatic protons and phosphorus.

Figure S6. ^{1}H **(A)** and ^{31}P NMR **(B)** spectra of 1-amino-2-(2,6-difluorophenyl)ethylphosphonic acid (**1g**).

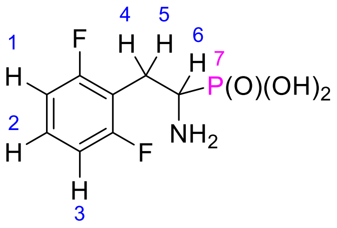

A

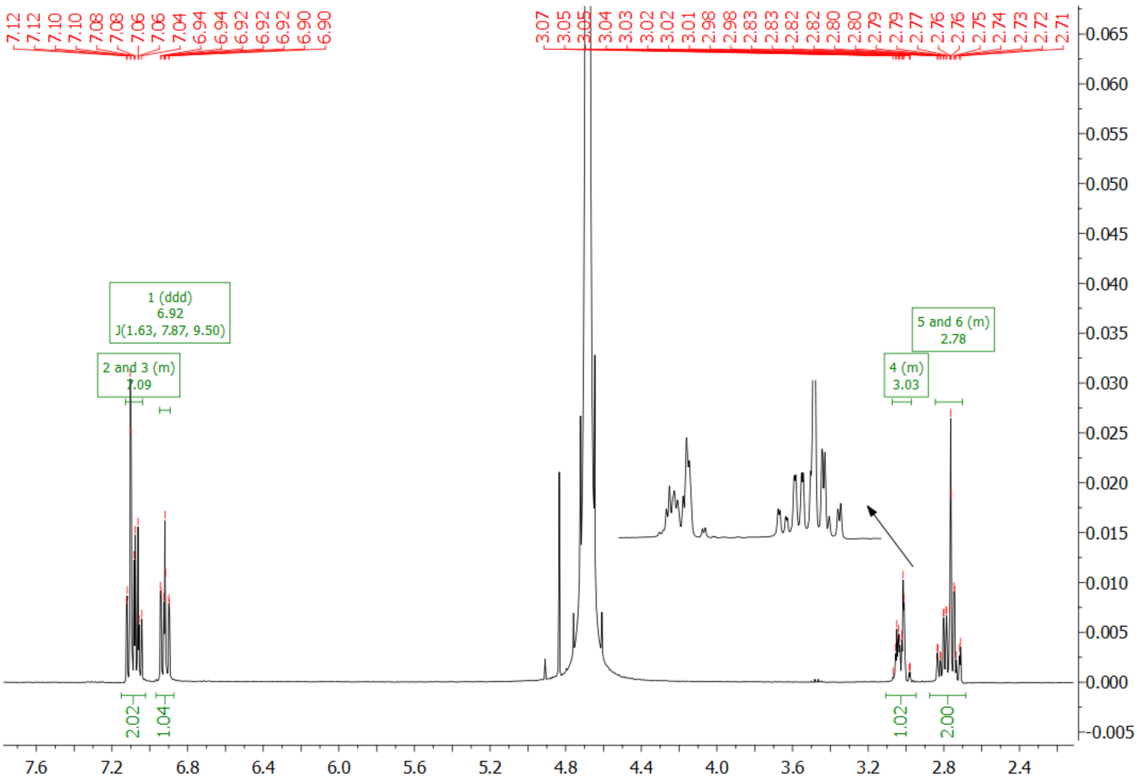

B

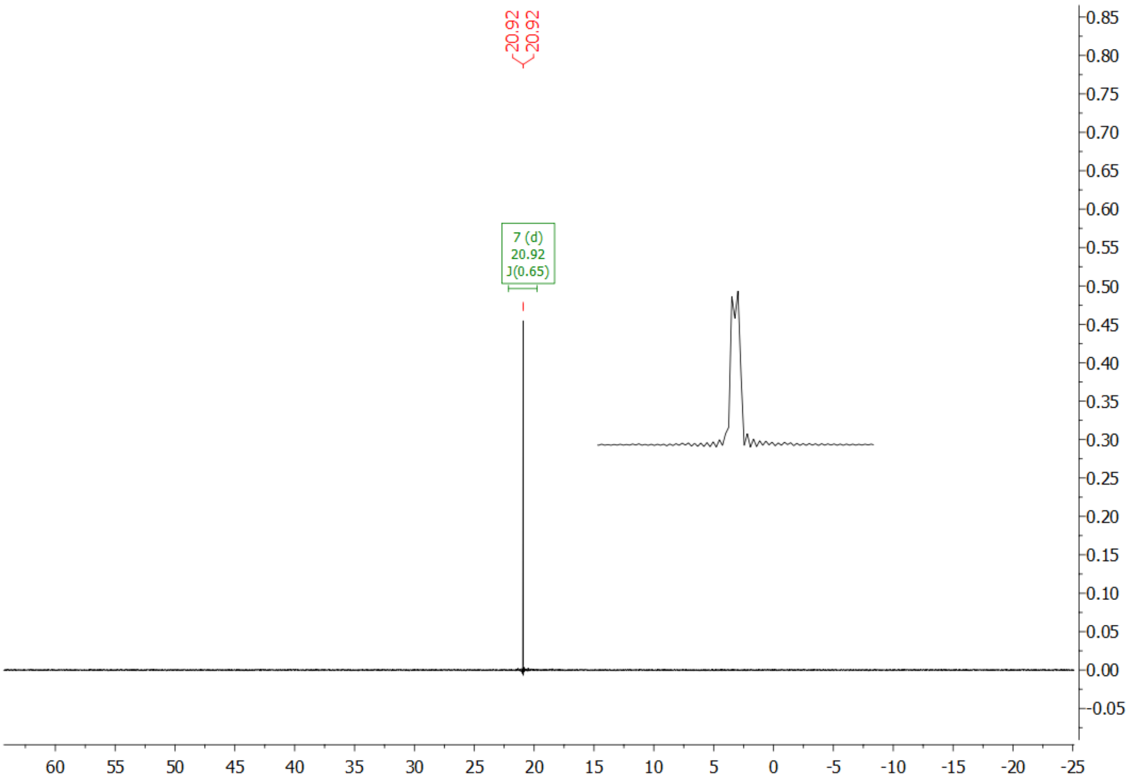

^{1}H NMR (400 MHz, D_2O+NaOD), (A) δ, ppm: 7.12 – 7.04 (m, 2H, 2xCH_{ar}), 6.92 (ddd, *J* = 9.5, 7.9, 1.6 Hz, 1H, CH_{ar}), 4.68 (br s, 1H, NH), 3.06 – 2.97 (m, 1H) and 2.84 – 2.71 (m, 2H, CH_2 and CHP)

^{31}P NMR (162 MHz, D_2O+NaOD), (B): δ, ppm: 20.92 (d, *J* = 0.6 Hz, 1P)

The distance between fluorine atoms and aliphatic protons is shorter than in other analogs and the resonance between them are very apparent through the deformations and overlapping of the peaks on the ^{1}H NMR spectra (Figure S6A). Only in this compound we obtained weak coupling (^{5}J = 0.6 Hz) between phosphorus atom and fluorine placed in *orto* position. It is a long distance coupling through 5 bonds visible on the ^{31}P NMR spectrum (Figure S6B).

Figure S7. Crystal structure of diethyl 1-N-formylamino-2-(4-fluorophenyl)ethylphosphonate (compound **7d**).

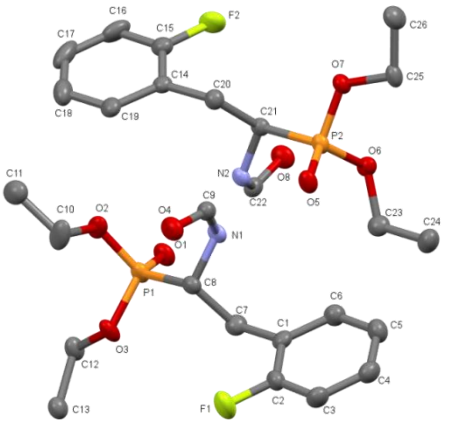

Figure S8. Modes of binding of both enantiomers of 1-amino-2-(3-chloro-4-fluorophenyl)ethylphosphonic acid (compound **1t**) to hAPN (**A**) and pAPN (**B**). Right hand side – R-isomer; left hand side – S-isomer. Coloration of the oxygen, amino, and fluorine atoms are identical as in Figure 1.

A

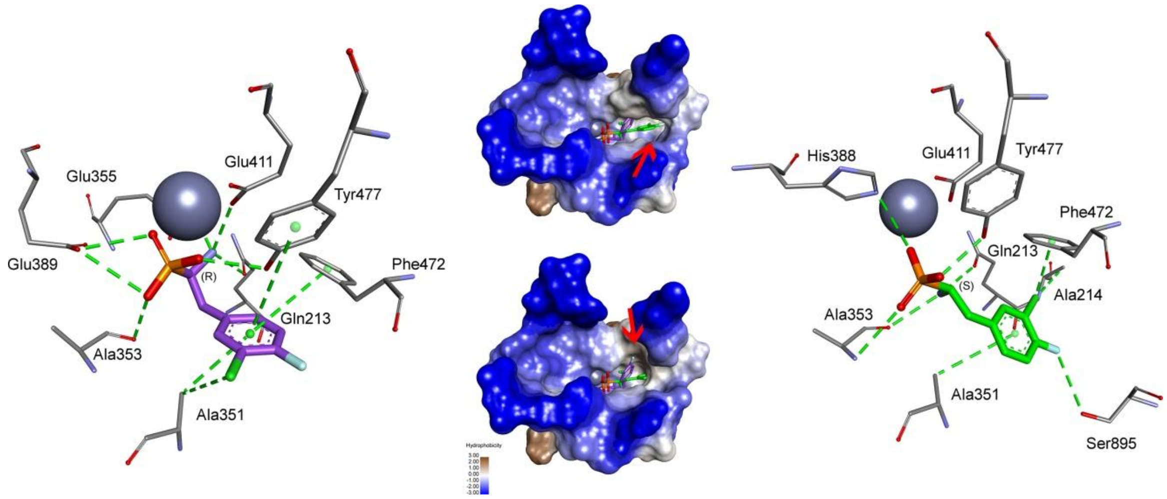

B

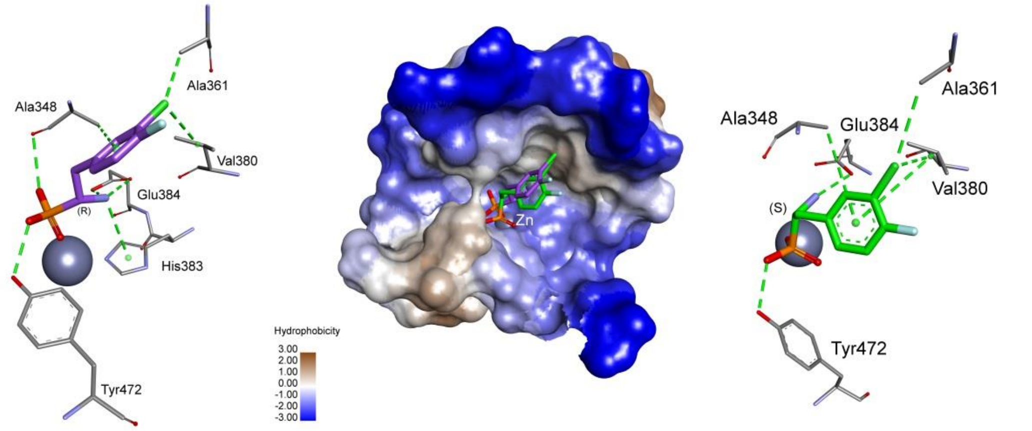

Figure S9. Modes of binding of both enantiomers of 1-amino-2-(2,6-difluorophenyl)ethylphosphonic acid (compound **1g**) to hAPN (**A**) and pAPN (**B**). Calculated internal hydrogen bonding between fluorine and amine nitrogen atoms in molecule **1g** (**C**). Right hand side – R-isomer; left hand side – S-isomer. Coloration of the oxygen, amino, and fluorine atoms are identical as in Figure 1.

A

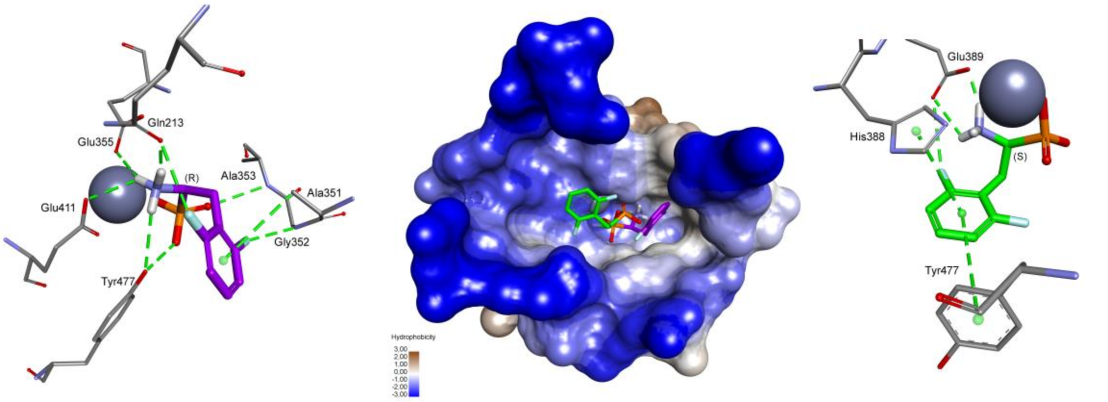

B

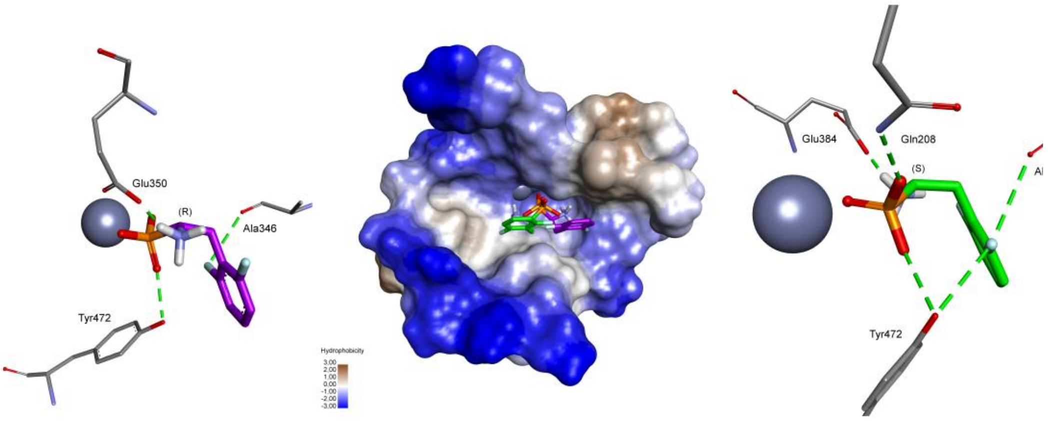

C

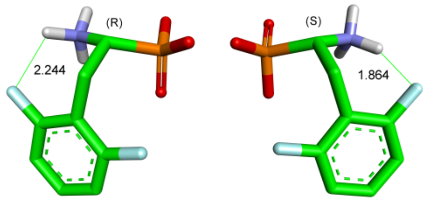

Supplementary Table S1 and S2.

Tables, which indicate the interactions between phosphonic acid analogs of phenylalanine and porcine aminopeptidase (pAPN) – Table S1 and human aminopeptidase (hAPN) – Table S2. The most important amino acid residues involved in coordination each of the inhibitors (for both enantiomeric forms) obtained after optimalization were marked in green color.

94 Table S1. Interactions of both isomers of compounds **1** with amino acids of porcine aminopeptidase (pAPN) taken for refinement during the IFD.

		Zn1035	Q206	Q208	S209	F344	N345	A346	G347	A348	M349	E350	Y357	R358	A:360	A361	L362	R376	V377	T379	V380	I381	H383	E384	H387	W399	L402	E406	A409	S410	E413	D434	S464	F467	Y472	S473
1a	S	Zn1035	Q206	Q208	S209	F344		A346	G347	A348	M349	E350	Y357	R358		A361		R376			V380		H383	E384	H387			E406		S410	E413		S464	F467	Y472	
1a	R	Zn1035		Q208		F344		A346	G347	A348	M349	E350	Y357	R358		A361		R376			V380		H383	E384	H387			E406						F467	Y472	
1b	S	Zn1035		Q208				A346	G347	A348	M349	E350	Y357	R358		A361		R376			V380		H383	E384	H387			E406		S410	E413			F467	Y472	
1b	R	Zn1035	Q206	Q208		F344		A346	G347	A348	M349	E350	Y357			A361		R376			V380		H383	E384	H387			E406			E413			F467	Y472	
1c	S	Zn1035		Q208		F344		A346	G347	A348	M349	E350		R358		A361		R376			V380		H383	E384	H387			E406		S410	E413			F467	Y472	
1c	R	Zn1035		Q208				A346	G347	A348	M349	E350	Y357	R358		A361		R376		T379	V380		H383	E384	H387			E406	A409	S410	E413			F467	Y472	
1d	S	Zn1035		Q208				A346	G347	A348	M349	E350	Y357	R358	A:360	A361		R376			V380		H383	E384	H387			E406		S410	E413			F467	Y472	
1d	R	Zn1035		Q208		F344		A346	G347	A348	M349	E350		R358		A361		R376			V380		H383	E384	H387			E406						F467	Y472	
1e	S	Zn1035				F344		A346	G347	A348	M349	E350	Y357	R358		A361		R376		T379	V380		H383	E384	H387			E406		S410	E413			F467	Y472	
1e	R	Zn1035					N345	A346	G347	A348	M349	E350	Y357	R358		A361		R376		T379	V380		H383	E384	H387			E406	A409	S410	E413			F467	Y472	
1f	S	Zn1035		Q208				A346	G347	A348	M349	E350									V380		H383	E384	H387			E406		S410				F467	Y472	
1f	R	Zn1035		Q208		F344		A346	G347	A348	M349	E350	Y357	R358		A361		R376			V380		H383	E384	H387			E406			E413			F467	Y472	
1g	S	Zn1035	Q206	Q208		F344		A346	G347	A348	M349	E350	Y357			A361				T379	V380		H383	E384	H387			E406		S410	E413			F467	Y472	
1g	R	Zn1035				F344		A346	G347	A348	M349	E350	Y357			A361		R376		T379	V380		H383	E384	H387			E406		S410	E413	D434		F467	Y472	S473
1h	S	Zn1035		Q208		F344		A346	G347	A348	M349	E350						R376			V380		H383	E384	H387			E406		S410	E413			F467	Y472	
1h	R	Zn1035		Q208				A346	G347	A348	M349	E350	Y357	R358		A361		R376			V380		H383	E384	H387			E406		S410	E413			F467	Y472	
1i	S	Zn1035		Q208				A346	G347	A348	M349	E350									V380		H383	E384	H387			E406			E413			F467	Y472	
1i	R	Zn1035		Q208			N345	A346	G347	A348	M349	E350	Y357	R358		A361		R376			V380		H383	E384	H387			E406		S410	E413			F467	Y472	
1j	S	Zn1035		Q208				A346	G347	A348	M349	E350	Y357	R358		A361		R376			V380		H383	E384	H387			E406		S410	E413			F467	Y472	
1j	R	Zn1035		Q208		F344		A346	G347	A348	M349	E350	Y357	R358		A361		R376			V380		H383	E384	H387			E406			E413			F467	Y472	
1k	S	Zn1035		Q208		F344	N345	A346	G347	A348	M349	E350		R358	A:360	A361		R376			V380		H383	E384	H387			E406						F467	Y472	
1k	R	Zn1035		Q208		F344		A346	G347	A348	M349	E350		R358		A361		R376			V380		H383	E384	H387			E406						F467	Y472	
1l	S	Zn1035		Q208				A346	G347	A348	M349	E350	Y357	R358		A361		R376		T379	V380		H383	E384	H387			E406		S410	E413			F467	Y472	
1l	R	Zn1035		Q208		F344		A346	G347	A348	M349	E350	Y357	R358		A361	L362	R376	V377		V380		H383	E384	H387			E406			E413			F467	Y472	
1m	S	Zn1035	Q206	Q208	S209	F344		A346	G347	A348	M349	E350	Y357	R358		A361		R376		T379	V380	I381	H383	E384	H387			E406		S410	E413		S464	F467	Y472	S473
1m	R	Zn1035	Q206	Q208				A346	G347	A348	M349	E350						R376		T379	V380		H383	E384	H387			E406	A409	S410	E413	D434		F467	Y472	
1n	S	Zn1035		Q208		F344		A346	G347	A348	M349	E350		R358		A361		R376			V380		H383	E384	H387			E406						F467	Y472	
1n	R	Zn1035		Q208		F344		A346	G347	A348	M349	E350				A361		R376			V380		H383	E384	H387			E406						F467	Y472	
1o	S	Zn1035		Q208		F344		A346	G347	A348	M349	E350	Y357	R358		A361		R376			V380		H383	E384	H387			E406		S410				F467	Y472	S473
1o	R	Zn1035					N345	A346	G347	A348	M349	E350	Y357	R358		A361		R376			V380		H383	E384	H387			E406		S410	E413	D434		F467	Y472	
1p	S	Zn1035	Q206	Q208	S209	F344		A346	G347	A348	M349	E350	Y357	R358		A361		R376			V380		H383	E384	H387			E406		S410	E413			F467	Y472	
1p	R	Zn1035		Q208		F344		A346	G347	A348	M349	E350				A361		R376			V380		H383	E384	H387			E406						F467	Y472	
1r	S	Zn1035		Q208		F344		A346	G347	A348	M349	E350		R358		A361		R376			V380		H383	E384	H387			E406						F467	Y472	
1r	R	Zn1035		Q208		F344		A346	G347	A348	M349	E350	Y357	R358		A361		R376			V380		H383	E384	H387			E406		S410	E413			F467	Y472	
1s	S	Zn1035		Q208		F344		A346	G347	A348	M349	E350		R358		A361		R376			V380		H383	E384	H387			E406						F467	Y472	
1s	R	Zn1035		Q208		F344		A346	G347	A348	M349	E350	Y357	R358		A361		R376			V380	I381	H383	E384	H387			E406						F467	Y472	
1t	S	Zn1035		Q208		F344		A346	G347	A348	M349	E350		R358		A361		R376			V380		H383	E384	H387			E406		S410	E413			F467	Y472	
1t	R	Zn1035				F344	N345	A346	G347	A348	M349	E350	Y357	R358	A:360	A361		R376			V380		H383	E384	H387			E406		S410	E413			F467	Y472	
1u	S	Zn1035		Q208				A346	G347	A348	M349	E350	Y357			A361				T379	V380		H383	E384	H387			E406		S410	E413			F467	Y472	
1u	R	Zn1035		Q208				A346	G347	A348	M349	E350	Y357	R358		A361		R376		T379	V380		H383	E384	H387			E406		S410	E413			F467	Y472	
1w	S	Zn1035		Q208				A346	G347	A348	M349	E350	Y357	R358		A361		R376			V380		H383	E384	H387			E406		S410	E413			F467	Y472	
1w	R	Zn1035		Q208		F344		A346	G347	A348	M349	E350	Y357	R358		A361		R376			V380		H383	E384	H387			E406						F467	Y472	
1v	S	Zn1035		Q208		F344		A346	G347	A348	M349	E350	Y357	R358		A361		R376			V380		H383	E384	H387			E406			E413			F467	Y472	
1v	R	Zn1035						A346	G347	A348	M349	E350	Y357	R358		A361		R376		T379	V380		H383	E384	H387			E406	A409	S410	E413			F467	Y472	
1x	S	Zn1035		Q208				A346	G347	A348	M349	E350	Y357	R358		A361		R376			V380		H383	E384	H387			E406		S410	E413			F467	Y472	
1x	R	Zn1035		Q208		F344		A346	G347	A348	M349	E350	Y357	R358		A361		R376			V380		H383	E384	H387			E406			E413			F467	Y472	
1y	S	Zn1035		Q208		F344		A346	G347	A348	M349	E350	Y357	R358		A361		R376			V380		H383	E384	H387			E406		S410				F467	Y472	
1y	R	Zn1035				F344		A346	G347	A348	M349	E350		R358		A361		R376		T379	V380	I381	H383	E384	H387			E406		S410	E413			F467	Y472	
1z	S	Zn1035		Q208				A346	G347	A348	M349	E350	Y357	R358		A361		R376			V380		H383	E384	H387			E406		S410	E413			F467	Y472	
1z	R	Zn1035		Q208			N345	A346	G347	A348	M349	E350	Y357	R358		A361		R376		T379	V380		H383	E384	H387			E406	A409	S410	E413			F467	Y472	
8	S	Zn1035	Q206	Q208	S209	F344		A346	G347	A348	M349	E350									V380		H383	E384	H387	W399	L402	E406					S464	F467	Y472	
8	R	Zn1035		Q208		F344		A346	G347	A348	M349	E350		R358		A361		R376			V380		H383	E384	H387			E406		S410				F467	Y472	

95 Table S2. Interactions of both isomers of compounds **1** with amino acids of human aminopeptidase (hAPN) taken for refinement furing the IFD.

		Zn1012	Q211	Q213	A214	F349	N350	A351	G352	A353	M354	E355	R363	S366	E380	R381	T384	V385	I386	H388	E389	H392	L407	E411	A414	S415	E418	Y419	D439	R442	S469	F472	Y477	S478	S895	F896	S897	N900
1a	S	Zn1012	Q211	Q213	A214	F349	N350	A351	G352	A353	M354	E355						V385		H388	E389	H392	L407	E411							S469	F472	Y477			F896	S897	
1a	R	Zn1012	Q211	Q213		F349	N350	A351	G352	A353	M354	E355						V385		H388	E389	H392		E411								F472	Y477			F896	S897	N900
1b	S	Zn1012	Q211	Q213		F349	N350	A351	G352	A353	M354	E355					T384	V385		H388	E389	H392		E411								F472	Y477		S895	F896	S897	
1b	R	Zn1012	Q211	Q213		F349		A351	G352	A353	M354	E355						V385		H388	E389	H392		E411								F472	Y477			F896	S897	
1c	S	Zn1012	Q211	Q213	A214	F349	N350	A351	G352	A353	M354	E355						V385		H388	E389	H392		E411							S469	F472	Y477			F896	S897	
1c	R	Zn1012	Q211	Q213	A214	F349	N350	A351	G352	A353	M354	E355						V385		H388	E389	H392		E411							S469	F472	Y477			F896	S897	
1d	S	Zn1012	Q211	Q213	A214	F349		A351	G352	A353	M354	E355						V385		H388	E389	H392		E411							S469	F472	Y477			F896	S897	
1d	R	Zn1012	Q211	Q213		F349	N350	A351	G352	A353	M354	E355						V385		H388	E389	H392		E411								F472	Y477		S895	F896	S897	N900
1e	S	Zn1012	Q211	Q213	A214	F349	N350	A351	G352	A353	M354	E355						V385		H388	E389	H392		E411							S469	F472	Y477			F896	S897	
1e	R	Zn1012		Q213			N350	A351	G352	A353	M354	E355		S366		R381	T384	V385		H388	E389	H392		E411	A414		E418						Y477					
1f	S	Zn1012	Q211	Q213		F349		A351	G352	A353	M354	E355				R381		V385		H388	E389	H392		E411							S469	F472	Y477			F896	S897	
1f	R	Zn1012	Q211	Q213		F349	N350	A351	G352	A353	M354	E355						V385		H388	E389	H392		E411								F472	Y477		S895	F896	S897	N900
1g	S	Zn1012	Q211	Q213	A214	F349	N350	A351	G352	A353	M354	E355						V385		H388	E389	H392		E411							S469	F472	Y477		S895	F896	S897	
1g	R	Zn1012		Q213				A351	G352	A353	M354	E355		S366		R381	T384	V385	I386	H388	E389	H392		E411	A414	S415	E418						Y477					
1h	S	Zn1012	Q211	Q213		F349	N350	A351	G352	A353	M354	E355						V385		H388	E389	H392	L407	E411							S469	F472	Y477			F896	S897	
1h	R	Zn1012	Q211	Q213	A214	F349	N350	A351	G352	A353	M354	E355						V385		H388	E389	H392	L407	E411							S469	F472	Y477			F896	S897	
1i	S	Zn1012		Q213				A351	G352	A353	M354	E355		S366	E380	R381	T384	V385		H388	E389	H392		E411	A414	S415	E418					F472	Y477					
1i	R	Zn1012	Q211	Q213	A214	F349	N350	A351	G352	A353	M354	E355						V385		H388	E389	H392		E411								F472	Y477			F896	S897	
1j	S	Zn1012						A351	G352	A353	M354	E355		S366	E380	R381	T384	V385		H388	E389	H392		E411	A414	S415	E418						Y477					
1j	R	Zn1012	Q211	Q213		F349		A351	G352	A353	M354	E355						V385		H388	E389	H392		E411								F472	Y477			F896	S897	
1k	S	Zn1012	Q211	Q213	A214	F349	N350	A351	G352	A353	M354	E355						V385		H388	E389	H392		E411							S469	F472	Y477			F896	S897	
1k	R	Zn1012	Q211	Q213		F349	N350	A351	G352	A353	M354	E355						V385		H388	E389	H392		E411								F472	Y477			F896	S897	N900
1l	S	Zn1012	Q211	Q213		F349	N350	A351	G352	A353	M354	E355	R363			R381		V385		H388	E389	H392		E411								F472	Y477		S895	F896	S897	N900
1l	R	Zn1012	Q211	Q213		F349	N350	A351	G352	A353	M354	E355						V385		H388	E389	H392		E411								F472	Y477		S895	F896	S897	
1m	S	Zn1012	Q211	Q213	A214	F349		A351	G352	A353	M354	E355						V385		H388	E389	H392	L407	E411							S469	F472	Y477			F896	S897	
1m	R	Zn1012		Q213				A351	G352	A353	M354	E355		S366		R381		V385		H388	E389	H392		E411	A414		E418					F472	Y477			F896		
1n	S	Zn1012		Q213			N350	A351	G352	A353	M354	E355	R363	S366		R381	T384	V385		H388	E389	H392		E411	A414	S415	E418						Y477			F896		
1n	R	Zn1012	Q211	Q213		F349	N350	A351	G352	A353	M354	E355						V385		H388	E389	H392		E411								F472	Y477			F896	S897	N900
1o	S	Zn1012	Q211	Q213		F349		A351	G352	A353	M354	E355						V385		H388	E389	H392		E411								F472	Y477			F896	S897	
1o	R	Zn1012		Q213				A351	G352	A353	M354	E355				R381	T384	V385		H388	E389	H392		E411	A414	S415	E418			R442		F472	Y477					
1p	S	Zn1012	Q211	Q213	A214	F349	N350	A351	G352	A353	M354	E355						V385		H388	E389	H392	L407	E411			E418				S469	F472	Y477			F896	S897	
1p	R	Zn1012		Q213				A351	G352	A353	M354	E355		S366		R381		V385		H388	E389	H392		E411	A414		E418					F472	Y477					
1r	S	Zn1012	Q211	Q213		F349	N350	A351	G352	A353	M354	E355						V385		H388	E389	H392		E411								F472	Y477		S895	F896	S897	
1r	R	Zn1012	Q211	Q213		F349	N350	A351	G352	A353	M354	E355						V385		H388	E389	H392		E411								F472	Y477		S895	F896	S897	N900
1s	S	Zn1012		Q213				A351	G352	A353	M354	E355				R381	T384	V385		H388	E389	H392		E411	A414	S415	E418			R442			Y477			F896		
1s	R	Zn1012		Q213				A351	G352	A353	M354	E355				R381	T384	V385		H388	E389	H392		E411	A414	S415	E418			R442			Y477			F896		
1t	S	Zn1012	Q211	Q213	A214	F349	N350	A351	G352	A353	M354	E355						V385		H388	E389	H392		E411							S469	F472	Y477		S895	F896	S897	
1t	R	Zn1012	Q211	Q213		F349	N350	A351	G352	A353	M354	E355						V385		H388	E389	H392		E411								F472	Y477		S895	F896	S897	
1u	S	Zn1012	Q211	Q213		F349	N350	A351	G352	A353	M354	E355						V385		H388	E389	H392		E411								F472	Y477		S895	F896	S897	
1u	R	Zn1012	Q211	Q213		F349	N350	A351	G352	A353	M354	E355						V385		H388	E389	H392		E411								F472	Y477			F896	S897	N900
1w	S	Zn1012						A351	G352	A353	M354	E355		S366		R381		V385		H388	E389	H392		E411	A414		E418						Y477					
1w	R	Zn1012		Q213				A351	G352	A353	M354	E355				R381	T384	V385		H388	E389	H392		E411	A414	S415	E418	Y419	D439	R442		F472	Y477	S478		F896		
1v	S	Zn1012	Q211	Q213		F349	N350	A351	G352	A353	M354	E355						V385		H388	E389	H392		E411								F472	Y477			F896	S897	N900
1v	R	Zn1012	Q211	Q213		F349	N350	A351	G352	A353	M354	E355						V385		H388	E389	H392		E411								F472	Y477			F896	S897	
1x	S	Zn1012	Q211	Q213		F349	N350	A351	G352	A353	M354	E355						V385		H388	E389	H392		E411								F472	Y477			F896	S897	N900
1x	R	Zn1012		Q213				A351	G352	A353	M354	E355		S366		R381		V385		H388	E389	H392		E411	A414		E418					F472	Y477					
1y	S	Zn1012						A351	G352	A353	M354	E355		S366		R381	T384	V385		H388	E389	H392		E411	A414		E418						Y477					
1y	R	Zn1012	Q211	Q213				A351	G352	A353	M354	E355		S366		R381		V385		H388	E389	H392		E411	A414		E418					F472	Y477			F896		
1z	S	Zn1012	Q211	Q213		F349		A351	G352	A353	M354	E355						V385		H388	E389	H392		E411							S469	F472	Y477			F896	S897	N900
1z	R	Zn1012		Q213				A351	G352	A353	M354	E355		S366		R381	T384	V385		H388	E389	H392		E411	A414	S415	E418			R442		F472	Y477			F896		
8	S	Zn1012	Q211	Q213	A214	F349		A351	G352	A353	M354	E355						V385		H388	E389	H392		E411							S469	F472	Y477			F896	S897	
8	R	Zn1012						A351	G352	A353	M354	E355				R381	T384	V385		H388	E389	H392		E411	A414	S415	E418						Y477					

Table S3. Crystal parameters and experimental details of the X-Ray data collection for structure **7d**

	**7d**
Crystal data	
Chemical formula	$C_{13}H_{19}FNO_4P$
M_r	303.26
Crystal system, space group	Triclinic, $P\bar{1}$
Temperature (K)	100.0(1)
a, *b*, *c* (Å)	8.4423 (4), 10.4112 (3), 17.1613 (7)
α, β, γ (°)	91.291 (3), 99.082 (4), 90.594 (3)
V (Å^3)	1488.95 (10)
Z	4
Radiation type	Mo *Kα*
μ (mm^{-1})	0.21
Crystal size (mm)	0.5 × 0.4 × 0.3
Data collection	
Diffractometer	Xcalibur
Absorption correction	–
No. of measured, independent and observed [$I > 2\sigma(I)$] reflections	10084, 5755, 3641
R_{int}	0.033
$(\sin \theta/\lambda)_{max}$ (Å^{-1})	0.617
Refinement	
$R[F^2 > 2\sigma(F^2)]$, $wR(F^2)$, S	0.040, 0.085, 0.86
No. of reflections	5755
No. of parameters	365
H-atom treatment	H-atom parameters constrained
$\Delta\rho_{max}$, $\Delta\rho_{min}$ (e Å^{-3})	0.34, −0.32

Table S4. Selected geometric parameters for crystal structure **7d** (Å, º)

P1—O1	1.4724 (14)	C17—C16	1.382 (3)
P1—O2	1.5648 (15)	C17—H17	0.9300
P1—O3	1.5724 (14)	C16—C15	1.367 (3)
P1—C8	1.810 (2)	C16—H16	0.9300
N1—C9	1.335 (3)	C15—C14	1.381 (3)
N1—C8	1.452 (2)	C7—C8	1.535 (3)
N1—H1	0.8600	C7—H7A	0.9700
F1—C2	1.357 (2)	C7—H7B	0.9700
C1—C2	1.378 (3)	O7—C25	1.462 (2)
C1—C6	1.388 (3)	C8—H8	0.9800
C1—C7	1.499 (3)	O8—C22	1.230 (2)
F2—C15	1.374 (2)	C9—H9	0.9300
P2—O5	1.4737 (13)	C11—H11A	0.9600
P2—O7	1.5660 (14)	C11—H11B	0.9600
P2—O6	1.5681 (14)	C11—H11C	0.9600

P2—C21	1.800 (2)	C20—C14	1.501 (3)
N2—C22	1.337 (2)	C20—C21	1.535 (3)
N2—C21	1.452 (2)	C20—H20A	0.9700
N2—H2	0.8600	C20—H20B	0.9700
O2—C10	1.464 (2)	C21—H21	0.9800
C2—C3	1.374 (3)	C22—H22	0.9300
C3—C4	1.377 (3)	C23—C24	1.500 (3)
C3—H3	0.9300	C23—H23A	0.9700
O3—C12	1.458 (2)	C23—H23B	0.9700
O4—C9	1.223 (2)	C24—H24A	0.9600
C4—C5	1.378 (3)	C24—H24B	0.9600
C4—H4	0.9300	C24—H24C	0.9600
C5—C6	1.382 (3)	C25—C26	1.490 (3)
C5—H5	0.9300	C25—H25A	0.9700
O6—C23	1.454 (2)	C25—H25B	0.9700
C6—H6	0.9300	C26—H26A	0.9600
C10—C11	1.471 (3)	C26—H26B	0.9600
C10—H10A	0.9700	C26—H26C	0.9600
C10—H10B	0.9700	C13—C12	1.497 (3)
C19—C18	1.374 (3)	C13—H13A	0.9600
C19—C14	1.389 (3)	C13—H13B	0.9600
C19—H19	0.9300	C13—H13C	0.9600
C18—C17	1.369 (3)	C12—H12A	0.9700
C18—H18	0.9300	C12—H12B	0.9700
O1—P1—O2	108.90 (8)	N1—C8—P1	111.36 (13)
O1—P1—O3	115.93 (8)	C7—C8—P1	108.46 (14)
O2—P1—O3	108.21 (8)	N1—C8—H8	108.6
O1—P1—C8	113.54 (9)	C7—C8—H8	108.6
O2—P1—C8	109.90 (9)	P1—C8—H8	108.6
O3—P1—C8	99.96 (8)	O4—C9—N1	125.4 (2)
C9—N1—C8	122.85 (16)	O4—C9—H9	117.3
C9—N1—H1	118.6	N1—C9—H9	117.3
C8—N1—H1	118.6	C10—C11—H11A	109.5
C2—C1—C6	116.0 (2)	C10—C11—H11B	109.5
C2—C1—C7	121.98 (19)	H11A—C11—H11B	109.5
C6—C1—C7	122.0 (2)	C10—C11—H11C	109.5
O5—P2—O7	116.86 (8)	H11A—C11—H11C	109.5
O5—P2—O6	113.24 (8)	H11B—C11—H11C	109.5
O7—P2—O6	103.52 (7)	C14—C20—C21	114.61 (17)
O5—P2—C21	113.73 (9)	C14—C20—H20A	108.6
O7—P2—C21	100.83 (9)	C21—C20—H20A	108.6
O6—P2—C21	107.35 (8)	C14—C20—H20B	108.6
C22—N2—C21	121.67 (16)	C21—C20—H20B	108.6
C22—N2—H2	119.2	H20A—C20—H20B	107.6
C21—N2—H2	119.2	N2—C21—C20	112.52 (16)
C10—O2—P1	123.68 (13)	N2—C21—P2	108.95 (13)

F1—C2—C3	118.19 (19)	C20—C21—P2	111.12 (13)
F1—C2—C1	117.67 (19)	N2—C21—H21	108.0
C3—C2—C1	124.1 (2)	C20—C21—H21	108.0
C2—C3—C4	118.4 (2)	P2—C21—H21	108.0
C2—C3—H3	120.8	O8—C22—N2	124.3 (2)
C4—C3—H3	120.8	O8—C22—H22	117.9
C12—O3—P1	122.22 (12)	N2—C22—H22	117.9
C3—C4—C5	119.7 (2)	O6—C23—C24	109.68 (17)
C3—C4—H4	120.2	O6—C23—H23A	109.7
C5—C4—H4	120.2	C24—C23—H23A	109.7
C4—C5—C6	120.4 (2)	O6—C23—H23B	109.7
C4—C5—H5	119.8	C24—C23—H23B	109.7
C6—C5—H5	119.8	H23A—C23—H23B	108.2
C23—O6—P2	122.71 (12)	C23—C24—H24A	109.5
C5—C6—C1	121.4 (2)	C23—C24—H24B	109.5
C5—C6—H6	119.3	H24A—C24—H24B	109.5
C1—C6—H6	119.3	C23—C24—H24C	109.5
O2—C10—C11	108.30 (17)	H24A—C24—H24C	109.5
O2—C10—H10A	110.0	H24B—C24—H24C	109.5
C11—C10—H10A	110.0	O7—C25—C26	108.09 (17)
O2—C10—H10B	110.0	O7—C25—H25A	110.1
C11—C10—H10B	110.0	C26—C25—H25A	110.1
H10A—C10—H10B	108.4	O7—C25—H25B	110.1
C18—C19—C14	121.5 (2)	C26—C25—H25B	110.1
C18—C19—H19	119.3	H25A—C25—H25B	108.4
C14—C19—H19	119.3	C25—C26—H26A	109.5
C17—C18—C19	120.5 (2)	C25—C26—H26B	109.5
C17—C18—H18	119.8	H26A—C26—H26B	109.5
C19—C18—H18	119.8	C25—C26—H26C	109.5
C18—C17—C16	120.2 (2)	H26A—C26—H26C	109.5
C18—C17—H17	119.9	H26B—C26—H26C	109.5
C16—C17—H17	119.9	C12—C13—H13A	109.5
C15—C16—C17	117.6 (2)	C12—C13—H13B	109.5
C15—C16—H16	121.2	H13A—C13—H13B	109.5
C17—C16—H16	121.2	C12—C13—H13C	109.5
C16—C15—F2	118.2 (2)	H13A—C13—H13C	109.5
C16—C15—C14	124.6 (2)	H13B—C13—H13C	109.5
F2—C15—C14	117.19 (19)	O3—C12—C13	107.68 (16)
C1—C7—C8	115.12 (18)	O3—C12—H12A	110.2
C1—C7—H7A	108.5	C13—C12—H12A	110.2
C8—C7—H7A	108.5	O3—C12—H12B	110.2
C1—C7—H7B	108.5	C13—C12—H12B	110.2
C8—C7—H7B	108.5	H12A—C12—H12B	108.5
H7A—C7—H7B	107.5	C15—C14—C19	115.65 (19)
C25—O7—P2	121.33 (12)	C15—C14—C20	122.86 (18)
N1—C8—C7	111.12 (16)	C19—C14—C20	121.48 (18)

O1—P1—O2—C10	171.34 (16)	C9—N1—C8—P1	108.21 (19)
O3—P1—O2—C10	44.52 (18)	C1—C7—C8—N1	57.4 (2)
C8—P1—O2—C10	−63.70 (18)	C1—C7—C8—P1	−179.86 (15)
C6—C1—C2—F1	179.41 (19)	O1—P1—C8—N1	70.46 (16)
C7—C1—C2—F1	−3.0 (3)	O2—P1—C8—N1	−51.78 (15)
C6—C1—C2—C3	−0.8 (3)	O3—P1—C8—N1	−165.42 (13)
C7—C1—C2—C3	176.8 (2)	O1—P1—C8—C7	−52.11 (16)
F1—C2—C3—C4	−179.7 (2)	O2—P1—C8—C7	−174.35 (12)
C1—C2—C3—C4	0.5 (4)	O3—P1—C8—C7	72.00 (15)
O1—P1—O3—C12	−39.76 (18)	C8—N1—C9—O4	−5.4 (3)
O2—P1—O3—C12	82.86 (16)	C22—N2—C21—C20	135.51 (18)
C8—P1—O3—C12	−162.20 (15)	C22—N2—C21—P2	−100.78 (18)
C2—C3—C4—C5	0.3 (3)	C14—C20—C21—N2	−57.0 (2)
C3—C4—C5—C6	−0.7 (4)	C14—C20—C21—P2	−179.52 (14)
O5—P2—O6—C23	24.93 (17)	O5—P2—C21—N2	−65.81 (15)
O7—P2—O6—C23	152.46 (14)	O7—P2—C21—N2	168.27 (12)
C21—P2—O6—C23	−101.43 (15)	O6—P2—C21—N2	60.27 (14)
C4—C5—C6—C1	0.4 (4)	O5—P2—C21—C20	58.71 (16)
C2—C1—C6—C5	0.3 (3)	O7—P2—C21—C20	−67.20 (14)
C7—C1—C6—C5	−177.3 (2)	O6—P2—C21—C20	−175.21 (13)
P1—O2—C10—C11	−163.45 (15)	C21—N2—C22—O8	2.8 (3)
C14—C19—C18—C17	1.0 (3)	P2—O6—C23—C24	−98.44 (18)
C19—C18—C17—C16	−0.3 (4)	P2—O7—C25—C26	−163.76 (13)
C18—C17—C16—C15	−0.6 (4)	P1—O3—C12—C13	173.53 (13)
C17—C16—C15—F2	−178.1 (2)	C16—C15—C14—C19	−0.4 (3)
C17—C16—C15—C14	1.0 (3)	F2—C15—C14—C19	178.73 (18)
C2—C1—C7—C8	81.0 (3)	C16—C15—C14—C20	−179.0 (2)
C6—C1—C7—C8	−101.5 (2)	F2—C15—C14—C20	0.2 (3)
O5—P2—O7—C25	61.77 (15)	C18—C19—C14—C15	−0.6 (3)
O6—P2—O7—C25	−63.45 (14)	C18—C19—C14—C20	178.0 (2)
C21—P2—O7—C25	−174.44 (14)	C21—C20—C14—C15	−62.2 (3)
C9—N1—C8—C7	−130.76 (19)	C21—C20—C14—C19	119.3 (2)

© 2020 by the authors. Submitted for possible open access publication under the terms and conditions of the Creative Commons Attribution (CC BY) license (http://creativecommons.org/licenses/by/4.0/).
