# Supplementary figures and images for: Phosphonic Acid Analogs of Fluorophenylalanines as Inhibitors of Human and Porcine Aminopeptidases N: Validation of the Importance of the Substitution of the Aromatic Ring
Source: Biomolecules. 2020 Apr 9;10(4):579. doi: 10.3390/biom10040579 (PMC7226027; doi:10.3390/biom10040579)

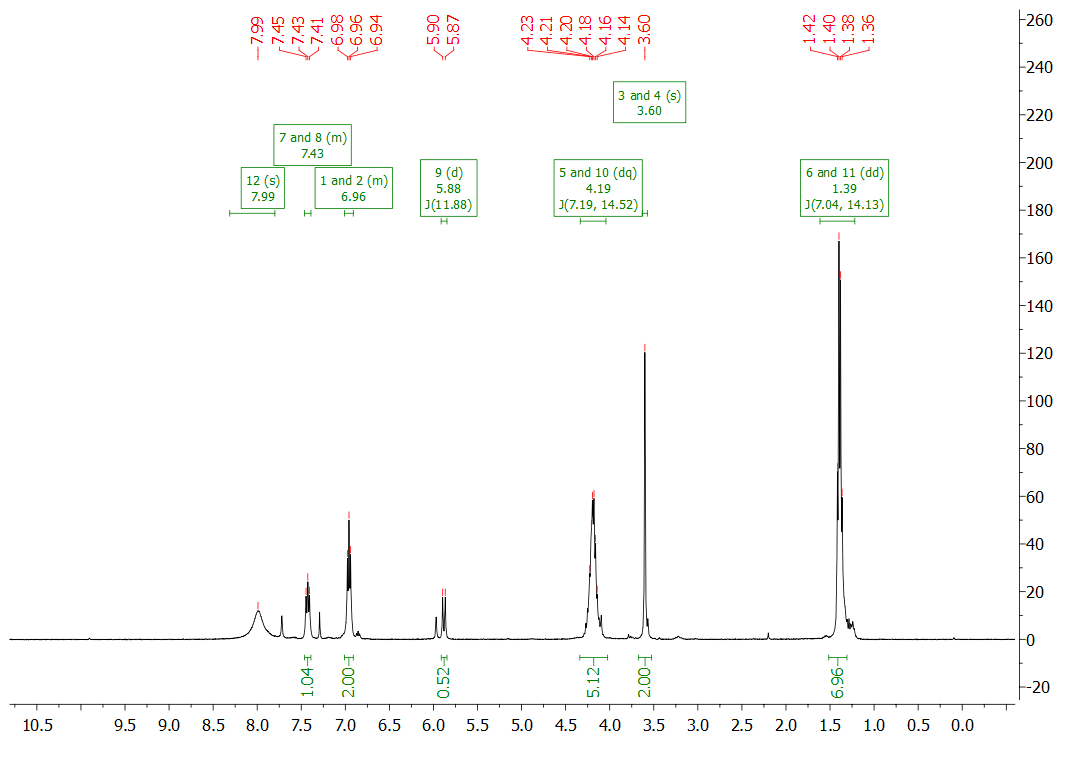

Supplement: Supplementary file 1 [file biomolecules-10-00579-s001.zip › Supplementary_Figures/Figure S1A.tiff]

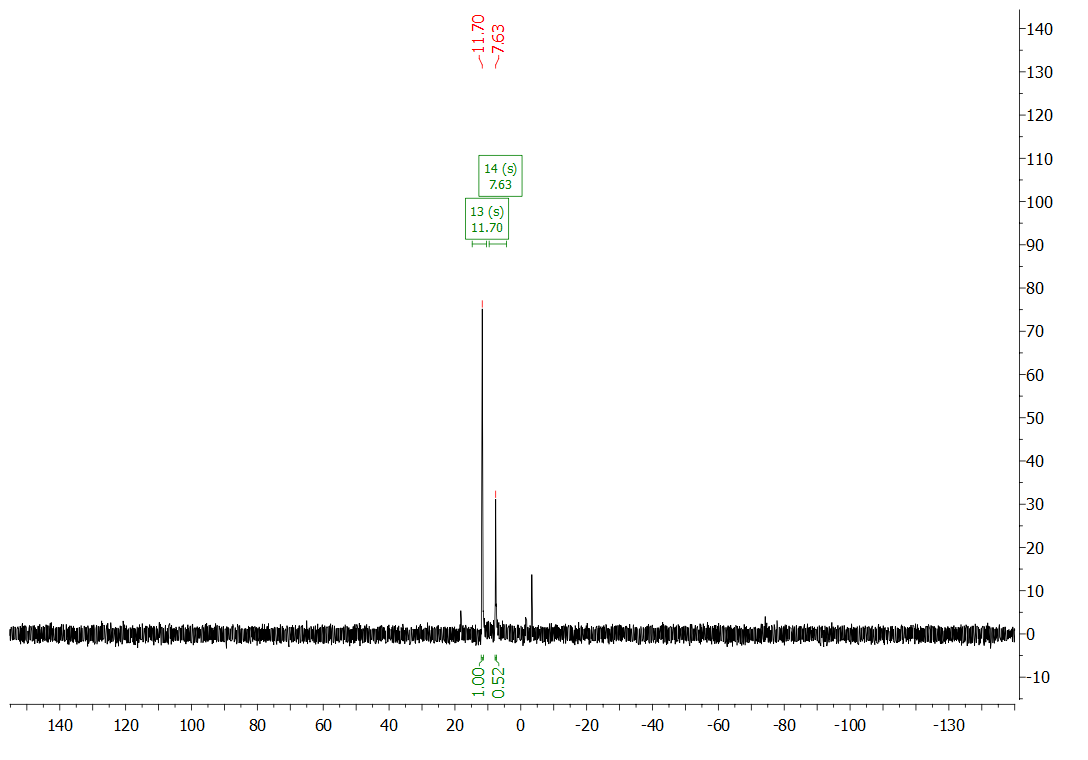

Supplement: Supplementary file 1 [file biomolecules-10-00579-s001.zip › Supplementary_Figures/Figure S1B.tiff]

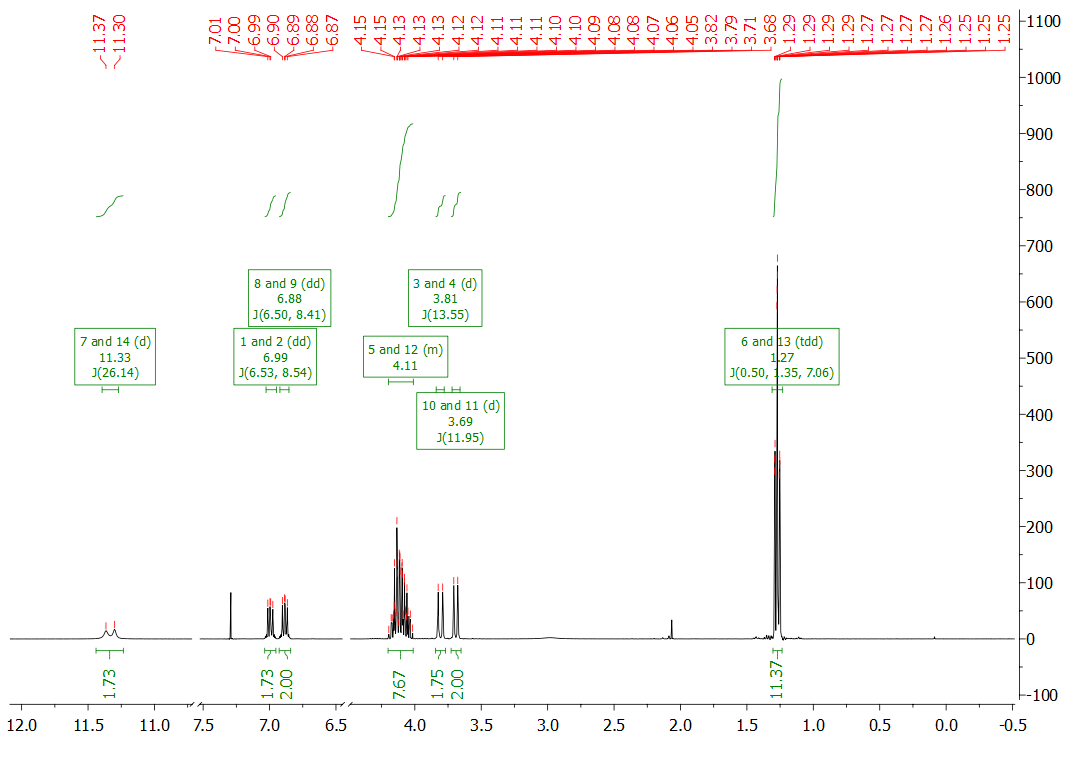

Supplement: Supplementary file 1 [file biomolecules-10-00579-s001.zip › Supplementary_Figures/Figure S2A.tiff]

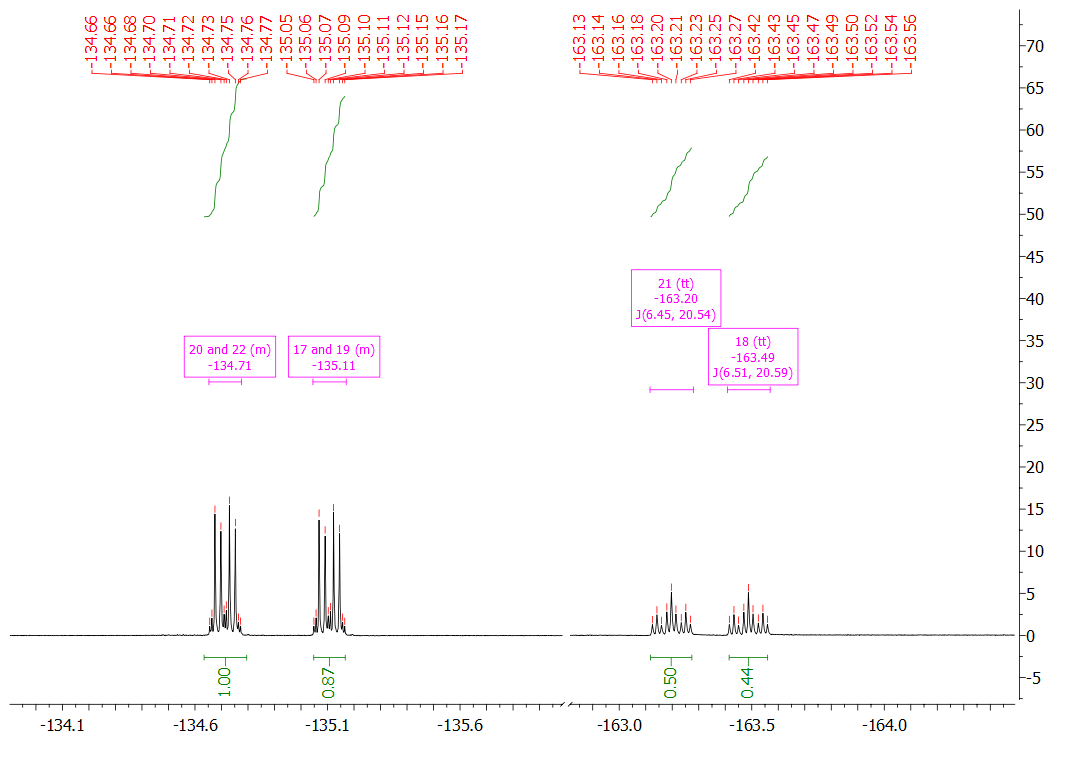

Supplement: Supplementary file 1 [file biomolecules-10-00579-s001.zip › Supplementary_Figures/Figure S2B.tiff]

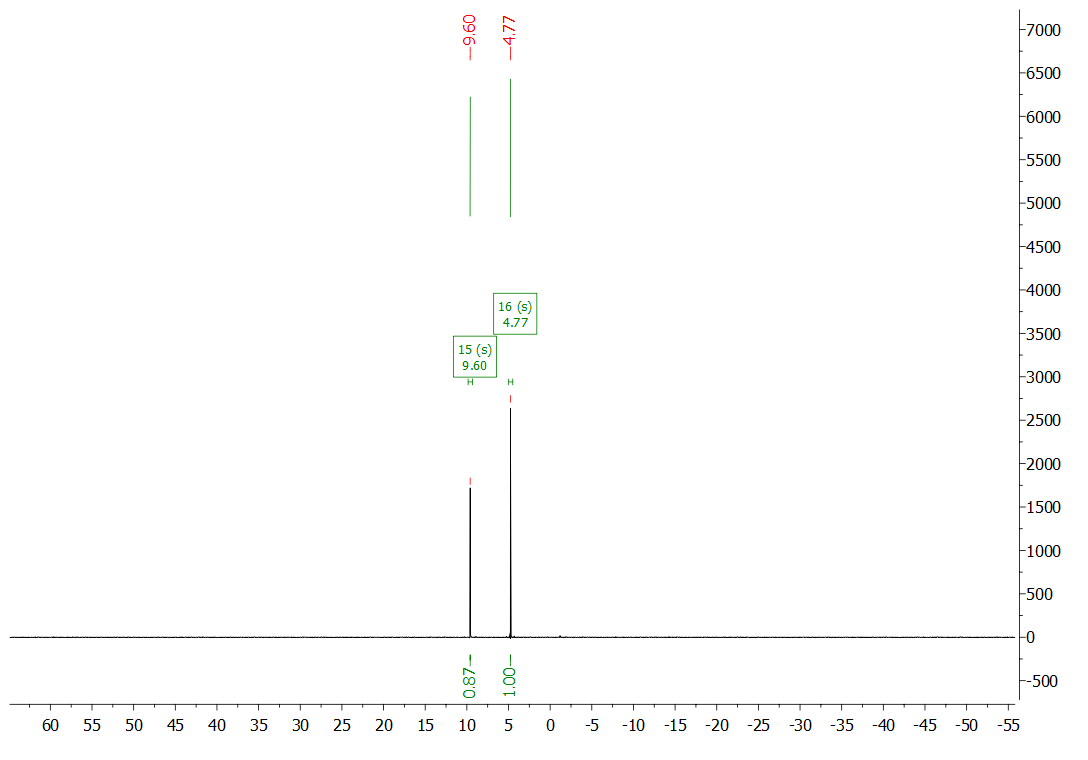

Supplement: Supplementary file 1 [file biomolecules-10-00579-s001.zip › Supplementary_Figures/Figure S2C.tiff]

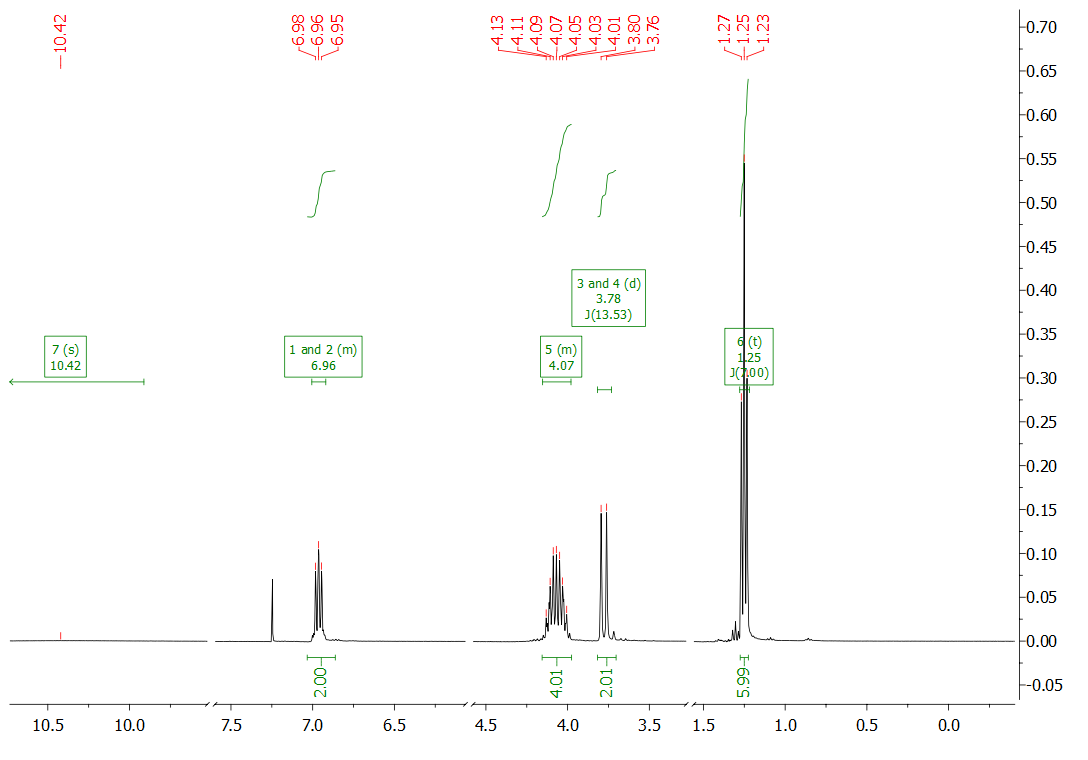

Supplement: Supplementary file 1 [file biomolecules-10-00579-s001.zip › Supplementary_Figures/Figure S3A.tiff]

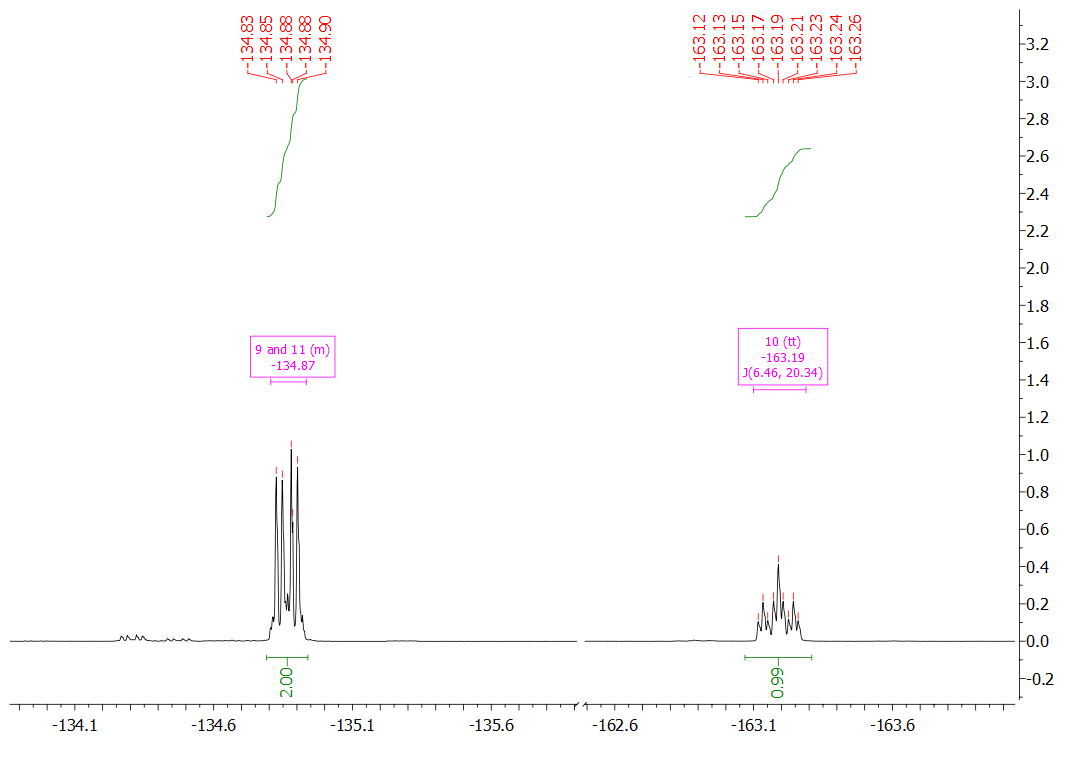

Supplement: Supplementary file 1 [file biomolecules-10-00579-s001.zip › Supplementary_Figures/Figure S3B.tiff]

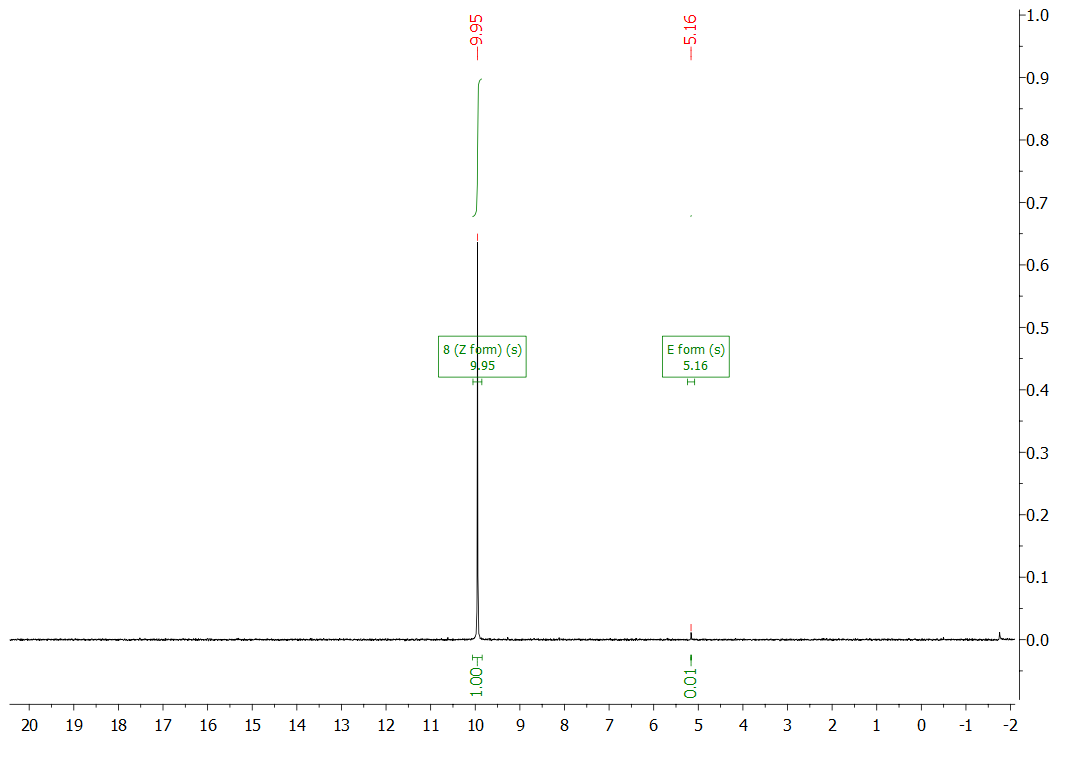

Supplement: Supplementary file 1 [file biomolecules-10-00579-s001.zip › Supplementary_Figures/Figure S3C.tiff]

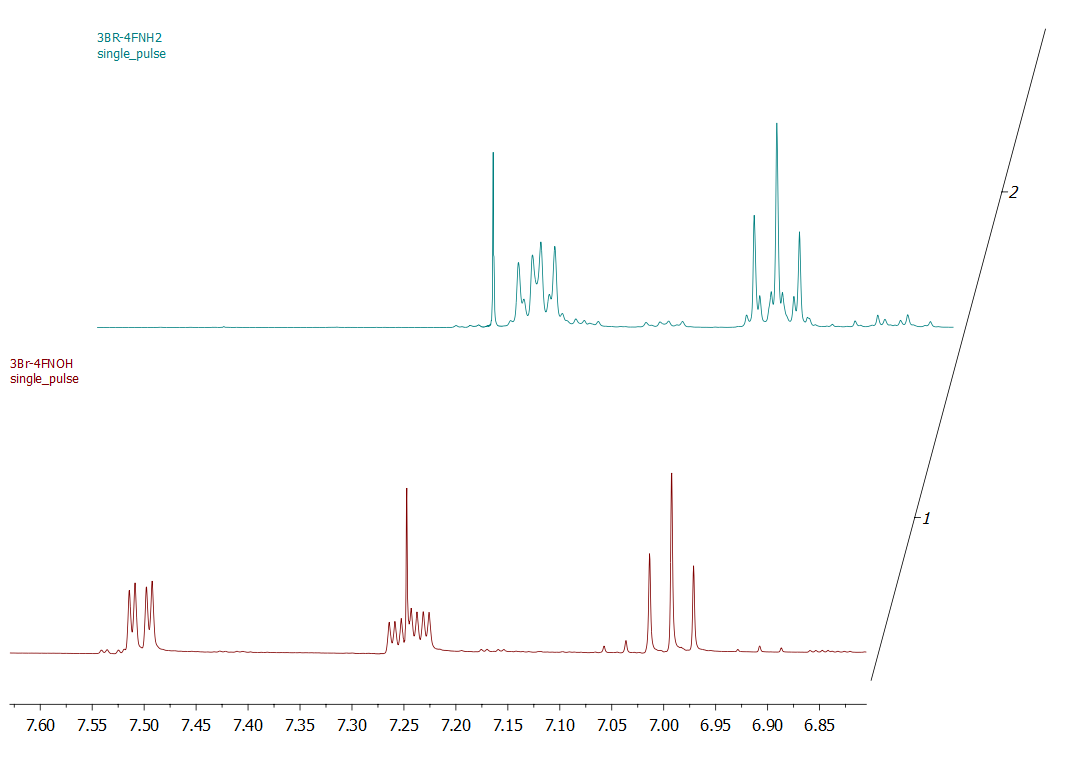

Supplement: Supplementary file 1 [file biomolecules-10-00579-s001.zip › Supplementary_Figures/Figure S4A.tiff]

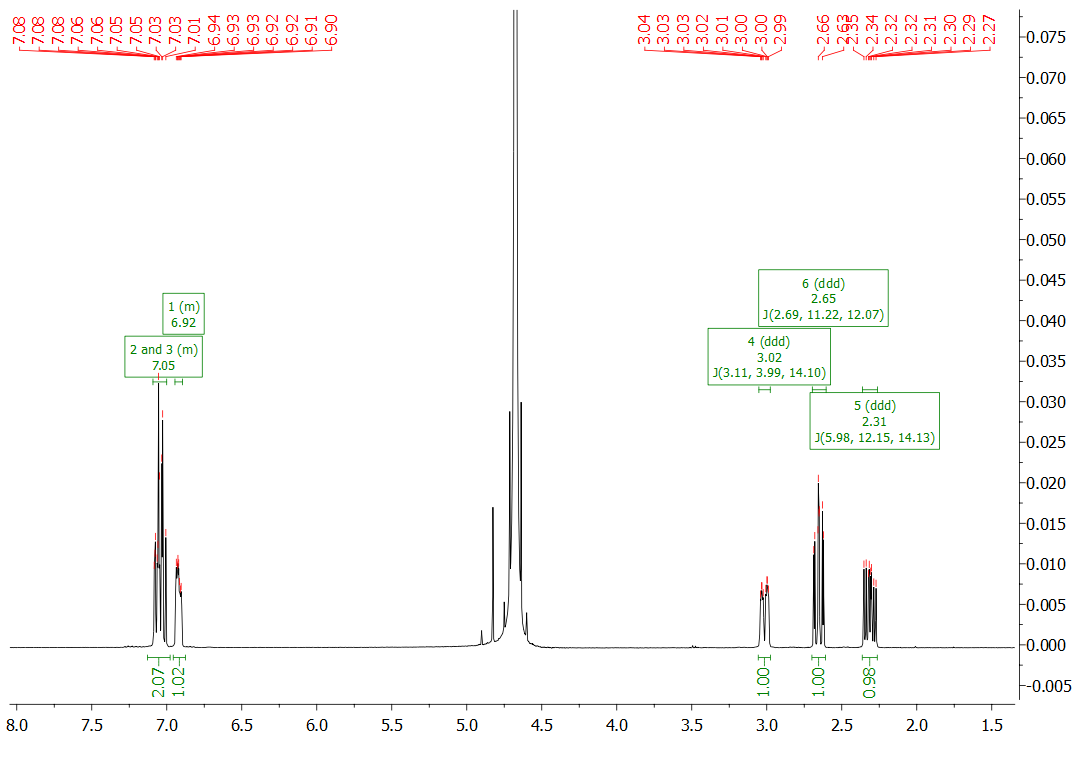

Supplement: Supplementary file 1 [file biomolecules-10-00579-s001.zip › Supplementary_Figures/Figure S5A.tiff]

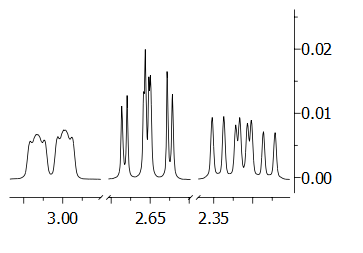

Supplement: Supplementary file 1 [file biomolecules-10-00579-s001.zip › Supplementary_Figures/Figure S5A_aliphatic region.tiff]

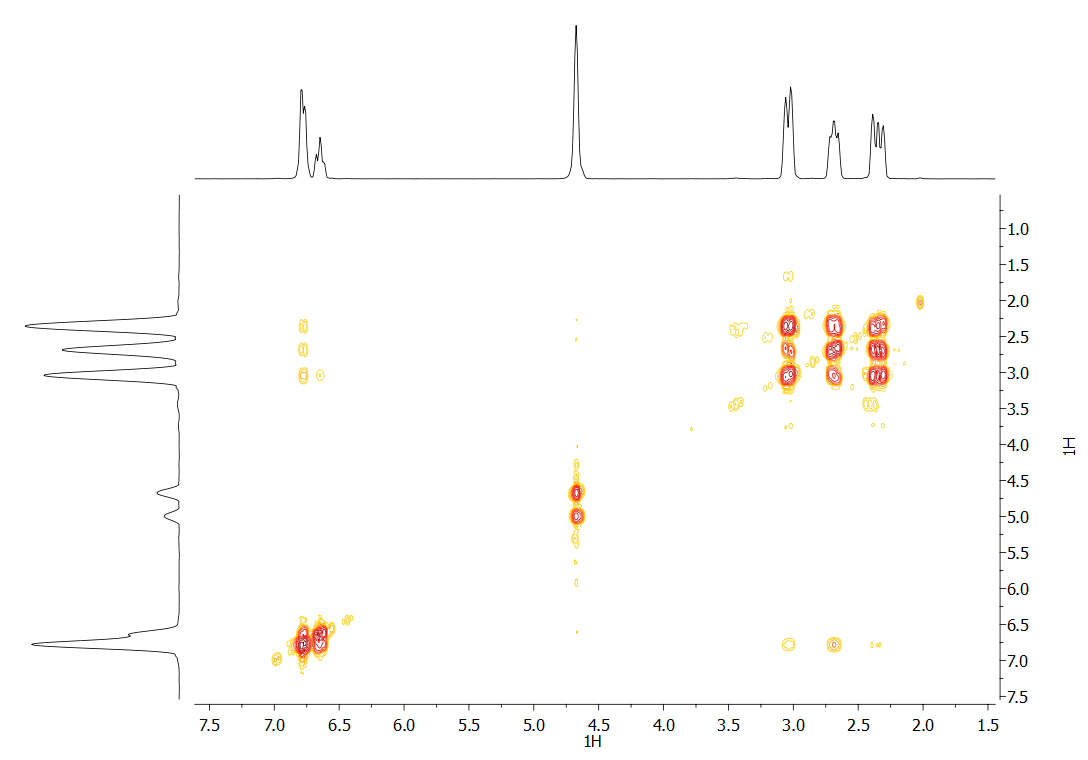

Supplement: Supplementary file 1 [file biomolecules-10-00579-s001.zip › Supplementary_Figures/Figure S5B.tiff]

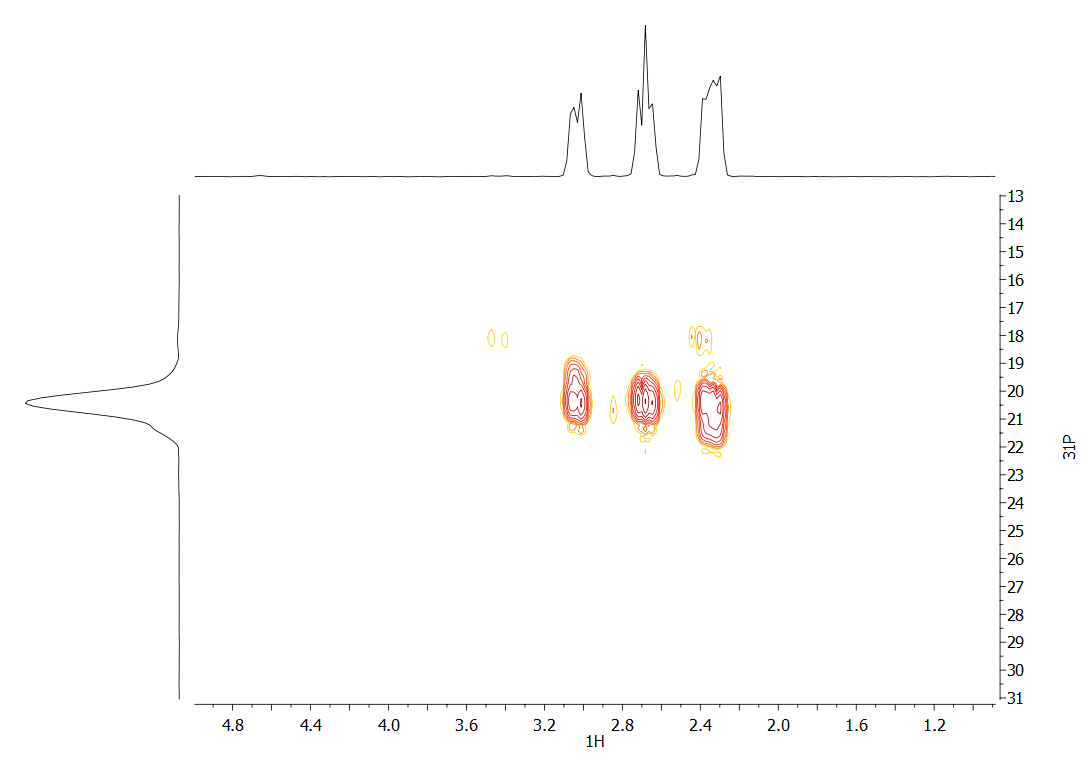

Supplement: Supplementary file 1 [file biomolecules-10-00579-s001.zip › Supplementary_Figures/Figure S5C.tiff]

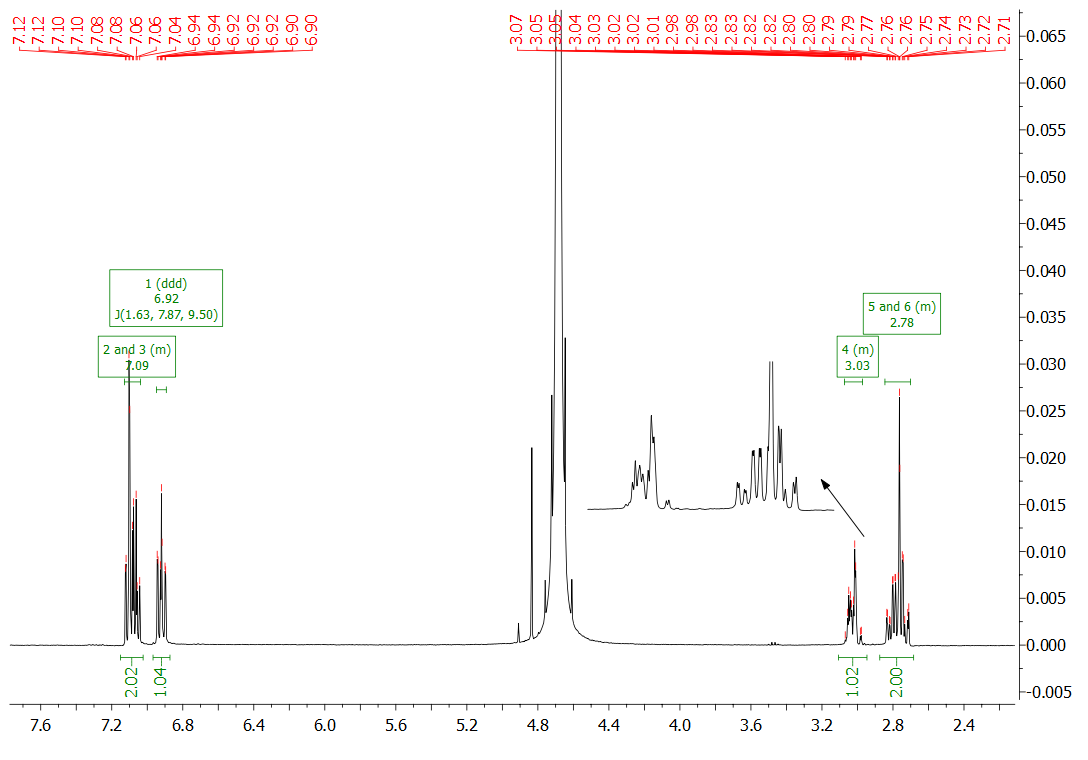

Supplement: Supplementary file 1 [file biomolecules-10-00579-s001.zip › Supplementary_Figures/Figure S6A.tiff]

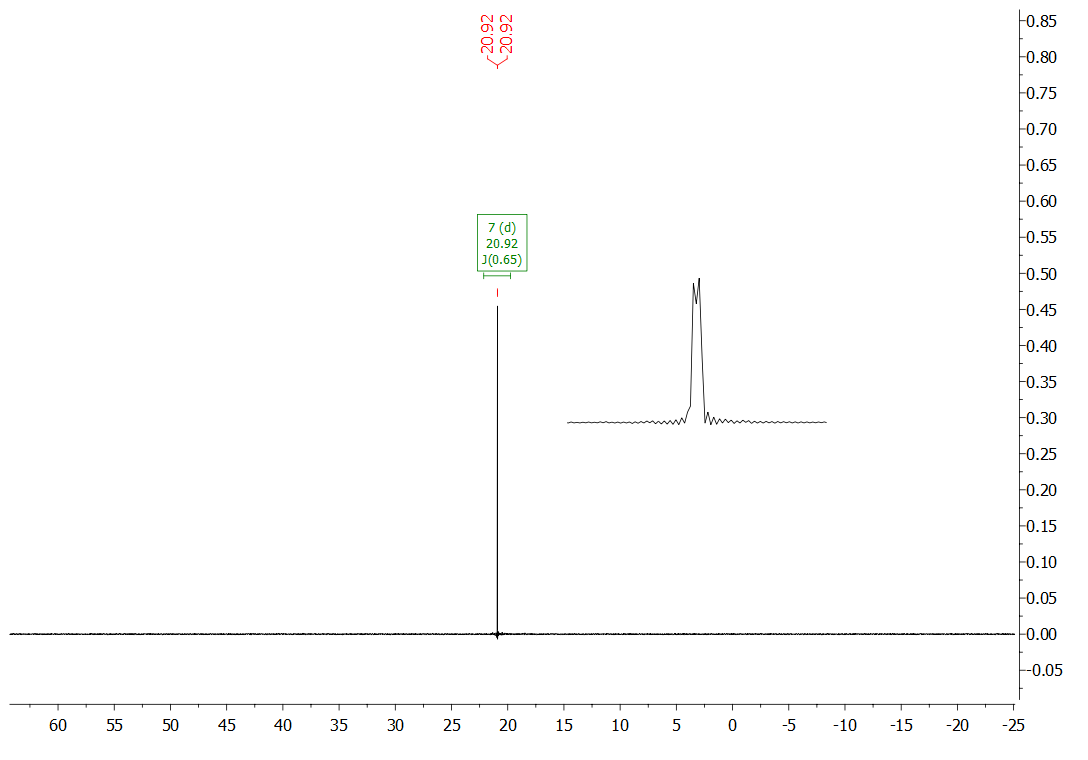

Supplement: Supplementary file 1 [file biomolecules-10-00579-s001.zip › Supplementary_Figures/Figure S6B.tiff]

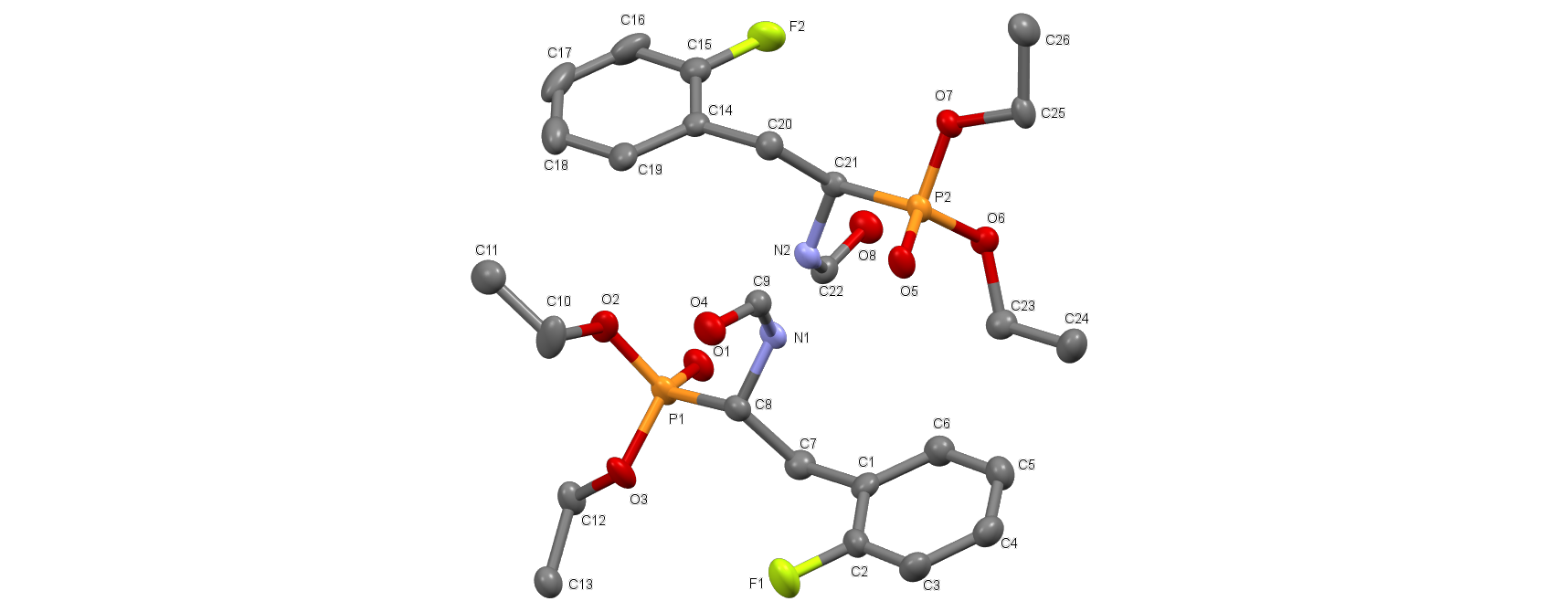

Supplement: Supplementary file 1 [file biomolecules-10-00579-s001.zip › Supplementary_Figures/Figure S7.tif]

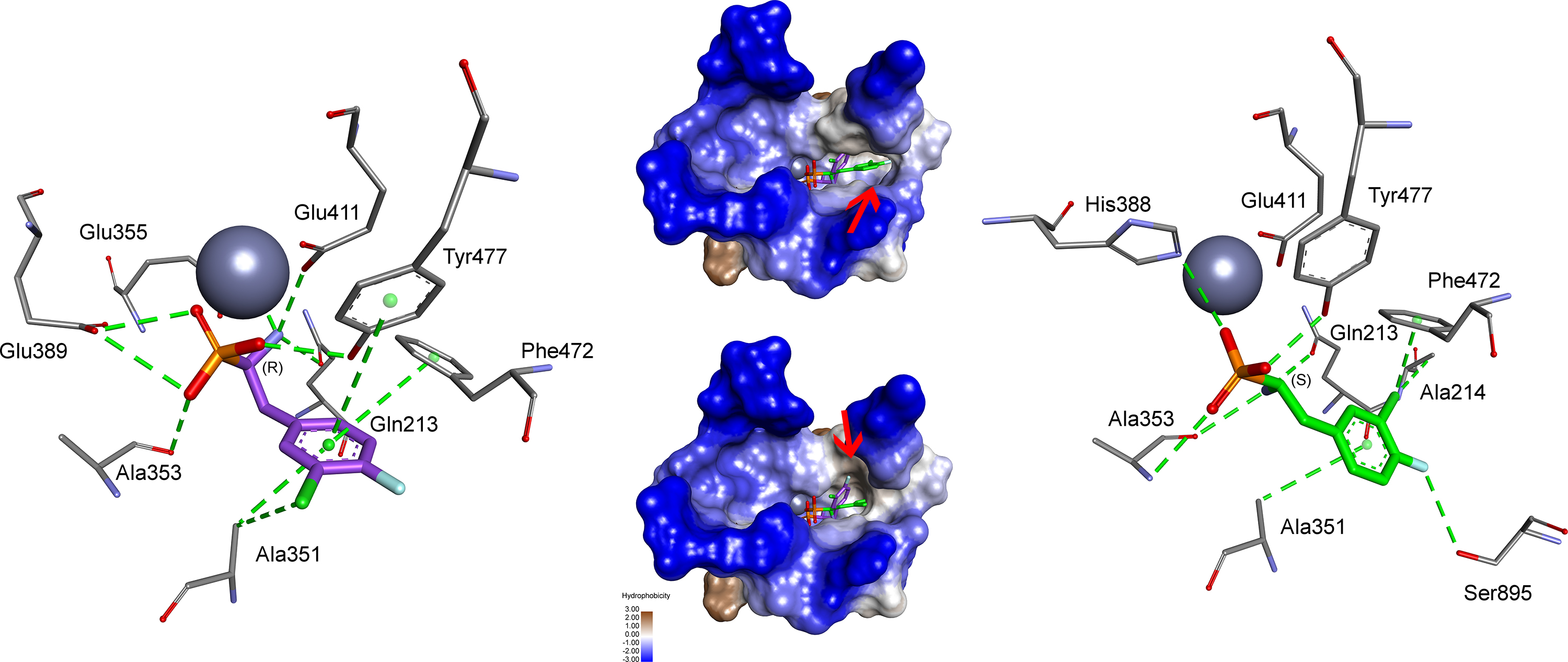

Supplement: Supplementary file 1 [file biomolecules-10-00579-s001.zip › Supplementary_Figures/Figure S8A.jpg]

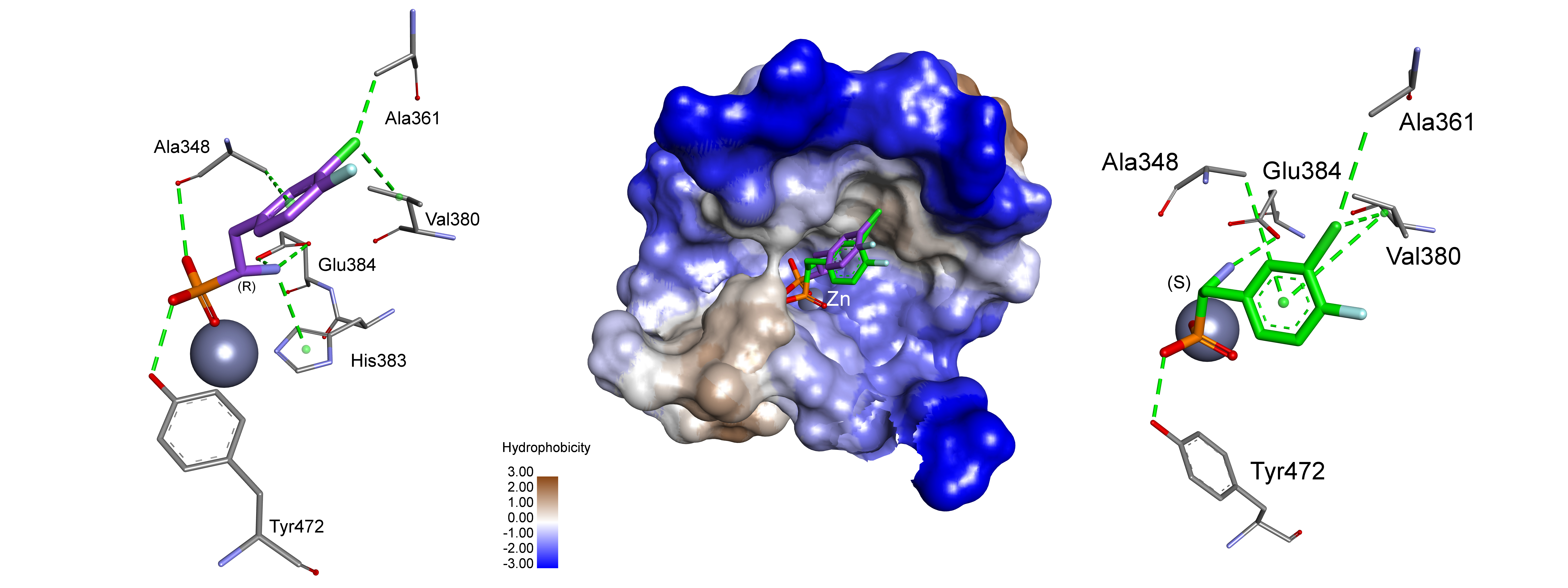

Supplement: Supplementary file 1 [file biomolecules-10-00579-s001.zip › Supplementary_Figures/Figure S8B.jpg]

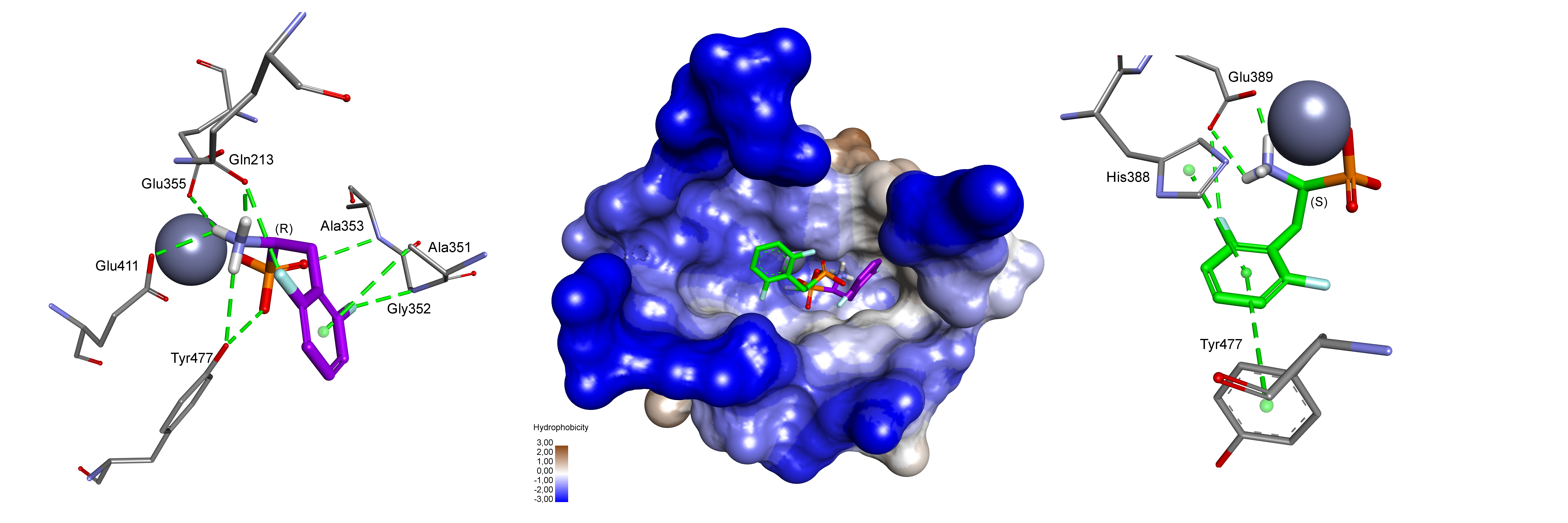

Supplement: Supplementary file 1 [file biomolecules-10-00579-s001.zip › Supplementary_Figures/Figure S9A.png]

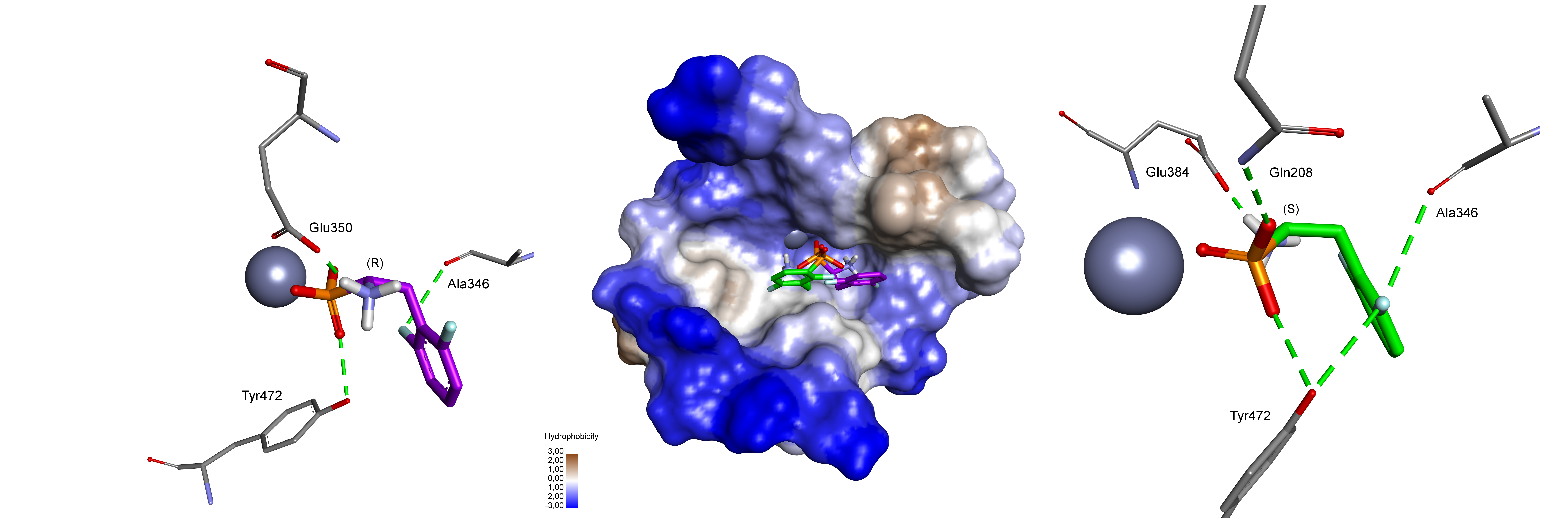

Supplement: Supplementary file 1 [file biomolecules-10-00579-s001.zip › Supplementary_Figures/Figure S9B.png]

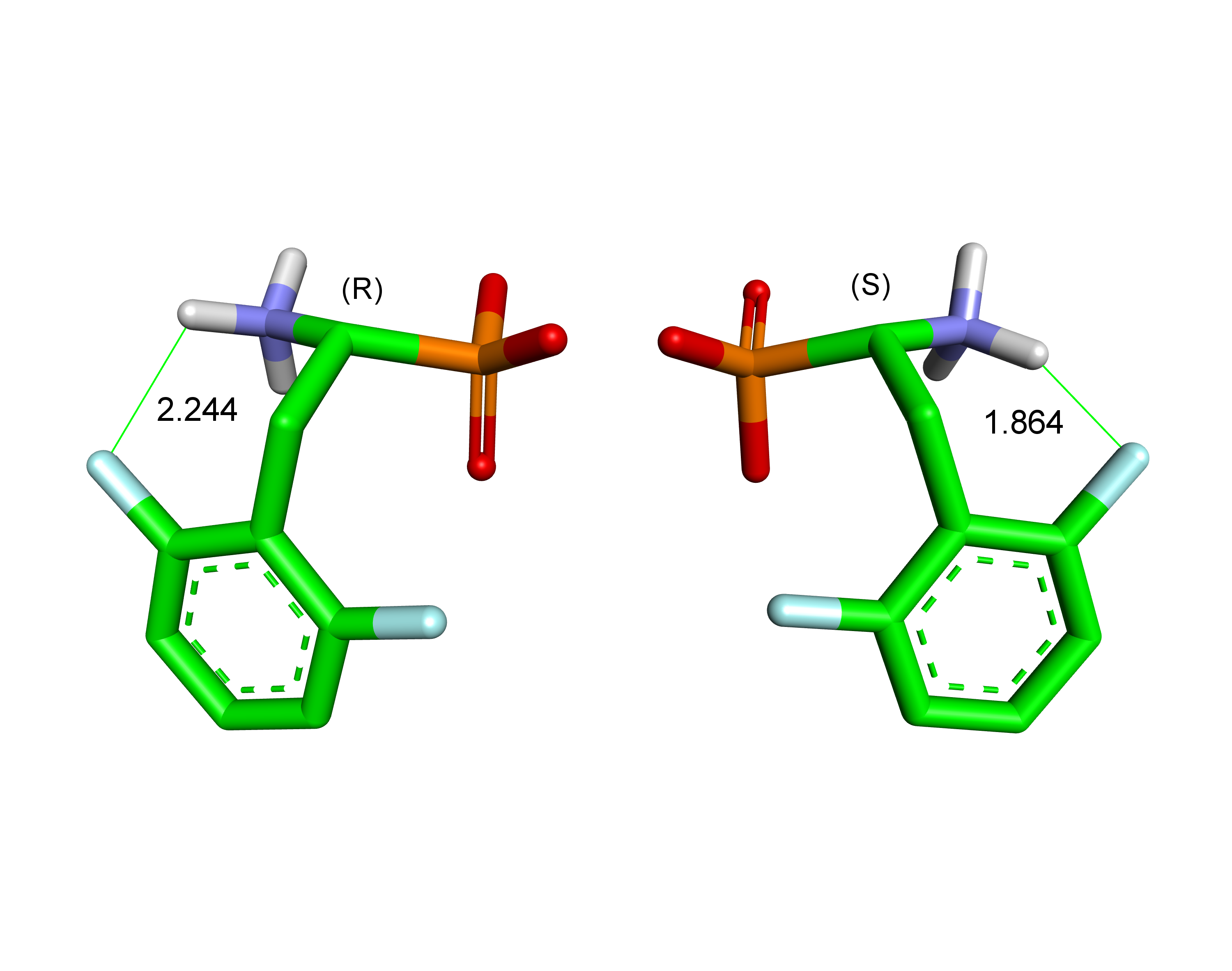

Supplement: Supplementary file 1 [file biomolecules-10-00579-s001.zip › Supplementary_Figures/Figure S9C.png]
